# Communication of anticancer drug benefits and related uncertainties to patients and clinicians: document analysis of regulated information on prescription drugs in Europe

**DOI:** 10.1136/bmj-2022-073711

**Published:** 2023-03-29

**Authors:** Courtney Davis, Anita K Wagner, Maximilian Salcher-Konrad, Henry Scowcroft, Barbara Mintzes, Adrian M J Pokorny, Jianhui Lew, Huseyin Naci

**Affiliations:** 1 Department of Global Health and Social Medicine, King’s College London, London, UK; 2 Department of Population Medicine, Harvard Medical School, and Harvard Pilgrim Health Care Institute, Boston, MA, USA; 3 Pharmacoeconomics Department, Austrian National Public Health Institute, Vienna, Austria; 4 Alzheimer’s Research UK, Cambridge, UK; 5 National Cancer Research Institute Bladder and Renal Research Group, London, UK; 6 School of Pharmacy, University of Sydney, Sydney, New South Wales, Australia; 7 Alice Springs Hospital, Northern Territory, Australia; 8 Department of Health Policy, London School of Economics and Political Science, London, UK

## Abstract

**Objective:**

To evaluate the frequency with which relevant and accurate information about the benefits and related uncertainties of anticancer drugs are communicated to patients and clinicians in regulated information sources in Europe.

**Design:**

Document content analysis.

**Setting:**

European Medicines Agency.

**Participants:**

Anticancer drugs granted a first marketing authorisation by the European Medicines Agency, 2017-19.

**Main outcome measures:**

Whether written information on a product addressed patients’ commonly asked questions about: who and what the drug is used for; how the drug was studied; types of drug benefit expected; and the extent of weak, uncertain, or missing evidence for drug benefits. Information on drug benefits in written sources for clinicians (summaries of product characteristics), patients (patient information leaflets), and the public (public summaries) was compared with information reported in regulatory assessment documents (European public assessment reports).

**Results:**

29 anticancer drugs that received a first marketing authorisation for 32 separate cancer indications in 2017-19 were included. General information about the drug (including information on approved indications and how the drug works) was frequently reported across regulated information sources aimed at both clinicians and patients. Nearly all summaries of product characteristics communicated full information to clinicians about the number and design of the main studies, the control arm (if any), study sample size, and primary measures of drug benefit. None of the patient information leaflets communicated information to patients about how drugs were studied. 31 (97%) summaries of product characteristics and 25 (78%) public summaries contained information about drug benefits that was accurate and consistent with information in regulatory assessment documents. The presence or absence of evidence that a drug extended survival was reported in 23 (72%) summaries of product characteristics and four (13%) public summaries. None of the patient information leaflets communicated information about the drug benefits that patients might expect based on study findings. Scientific concerns about the reliability of evidence on drug benefits, which were raised by European regulatory assessors for almost all drugs in the study sample, were rarely communicated to clinicians, patients, or the public.

**Conclusions:**

The findings of this study highlight the need to improve the communication of the benefits and related uncertainties of anticancer drugs in regulated information sources in Europe to support evidence informed decision making by patients and their clinicians.

## Introduction

To receive and participate in medical care, patients need high quality information about treatments, tests, and services—including information about the benefits of and risks from prescription drugs.^1^ ^2^ Provision of information can support ethical principles of patient autonomy and informed consent, facilitate shared decision making, and help to ensure that treatment is sensitive to, and meets the needs and priorities of, individuals.^3^ ^4^ ^5^ ^6^ ^7^ Patients value high quality, written information to supplement and reinforce the verbal information given by clinicians.^3^ ^8^ ^9^ ^10^ ^11^ ^12^ ^13^ ^14^ ^15^ ^16^ ^17^ ^18^ ^19^ ^20^ ^21^ ^22^ ^23^ This is the case even for those who do not want to participate in shared decision making.^9^ ^12^

Patients can access information on prescription drugs from many sources, but much of this information is unregulated and has not been evaluated, and it may not be of good quality.^3^ ^11^ ^20^ ^24^ Information on prescription drugs should consider patients’ needs and expectations and address patient relevant questions. The information should also be up to date and consistent with the available evidence, while also acknowledging uncertainties such as weak or missing evidence. All pertinent information needed for informed decision making should be available, including details of relevant evidence gaps and uncertainties.^3^ ^11^ ^20^ ^24^ ^25^

Because drug regulatory agencies conduct independent, critical appraisals of the evidence supporting new drug approvals, in principle they are well placed to develop and disseminate high quality information to patients.^10^ ^18^ ^21^ ^26^ ^27^ Additionally, in the United Kingdom and European Union it is mandatory for all approved medicines to be accompanied by written (including electronic) information for patients and healthcare professionals (see box 1) that has been approved or produced by the Medicines and Healthcare products Regulatory Agency (MHRA) in the UK or the European Medicines Agency (EMA) in the EU. The content, format, and structure of the information for clinicians and patients are strictly governed by statute and regulatory templates. Information about a drug’s side effects and potential harms must be included in patient leaflets. Although information about a drug’s benefits is not required by EU or UK legislation, it is permitted if it is non-promotional and consistent with the information provided to clinicians (see box 2).

Box 1 **Written medicines information products for doctors, patients, and the public in the UK and EU**

**Summary of product characteristics for healthcare professionals**

The summary of product characteristics is disseminated for each medical product approved for use in the EU is the primary way manufacturers and medicines regulators communicate comprehensive and essential information for use of a drug to healthcare professionals (equivalent to prescription drug labelling in the United States). The summary of product characteristics is drafted by the drug manufacturer (in line with current legislation and guidance on content and layout) but must be approved by regulatory agencies. Summaries of product characteristics are also available in electronic format from the electronic Medicines Compendium and from the websites of the European Medicines Agency (EMA) and the UK’s Medicines and Healthcare products Regulatory Agency (MHRA).

**Package leaflets for patients**

The package leaflet for patients is also referred to as the patient information leaflet. The content of each package leaflet must be drawn up in accordance with a drug’s official summary of product characteristics. As with summaries of product characteristics, package leaflets are drafted by the drug manufacturer and approved by regulatory agencies. Since 2004, EU legislation has required that all leaflets must be user tested for readability. Statutory patient information leaflets are the most widely available source of printed information on medicines in the UK and EU, because pharmaceutical companies are required to include them in every medicine pack. Patient information leaflets are also available in electronic format from the electronic Medicines Compendium and from the websites of EMA and MHRA.

The summary of product characteristics and the package leaflet are collectively referred to by EMA as the product information. In addition to the official product information, which is drafted by the manufacturer and approved by EMA, a summary of the European Public Assessment Report (EPAR) is produced by EMA for the lay public (also called a medicine overview).

**EPAR summaries for the public**

Since 2004, EU legislation has required that EMA produce a lay summary of the EPAR for each medicinal product marketed in the EU. EPARs are the publicly available documents accompanying each medicine granted (or refused) a marketing authorisation by EMA. EPARs detail regulators’ scientific deliberations throughout the regulatory assessment process and the conclusions they reach at its end. While the full EPAR reports are lengthy and mainly written for professionals, EPAR summaries must be written in a language that is understandable to the public and translated into all official EU languages. These public summaries are available in electronic format from EMA’s website and include information on a drug’s benefits and harms, based on evidence from clinical studies evaluated as part of EMA’s assessment procedure. Public summaries are drafted by EMA medical writers and are reviewed by EMA assessors and EMA patient representatives.

The MHRA also produces lay summaries of its public assessment reports. These are available on the MHRA website.

Box 2 European Medicines Agency guidance on including benefit information in the package leaflet for patients*

**Information on the benefits of using this medicine**

On a case-by-case basis, information on the benefits of the treatment could be included in this section, as long as it is compatible with the summary of product characteristics, useful for the patient, and to the exclusion of any element of a promotional nature (in accordance with art 62 of Directive 2001/83/EC). This could be included under a separate subheading (eg, entitled How X works). The information should be depicted in a clear and condensed way. For example, information could relate to:

Signs and symptoms of the target disease, in particular for non-prescription medicines, but also for medicines to be taken on demand (eg, treatment of migraine);
The benefits of taking the medicine could be summarised (eg, this medicine reduces pain associated with arthritis, this medicine has been shown to reduce blood sugar, which helps to prevent complications from your diabetes). This would be particularly important to encourage adherence to the treatment (eg, for long term and prevention treatment). Benefit may be described in terms of prevention of disease complications (eg, antidiabetes), if established. The timing of the effect may also be described if useful. In any case, information must be compatible with the summary of product characteristics, in particular section 5.1;
Information on the amount of time the medicine usually takes to work may be presented if relevant for the patient (eg, painkiller, antidepressant).

*From EMA’s Quality Review of Documents template (version 10.3 09/2022).

One clear example of how high quality information is fundamental to patient centred care is in the specialty of cancer. Patients with advanced, non-curable cancer can face particularly difficult decisions as they must often weigh a small, or even unknown, increase in survival time against the toxicity of treatment.^12^ ^28^ ^29^ ^30^ ^31^ In addition, there is often considerable uncertainty around the balance of harms and benefits for new anticancer drugs when they enter the market, and evidence on patient relevant outcomes, such as overall survival and quality of life, is often missing.^32^ ^33^ ^34^ ^35^ ^36^ However, prescribers and patients may be unaware of evidence gaps and uncertainties, and they often have unrealistic expectations of drug benefit.^37^ ^38^ ^39^ ^40^ ^41^ ^42^ ^43^ Since patients’ tolerance for risk of harm or uncertainty of benefit varies between individuals, it is critical that patients are given full information about the benefits, harms, and uncertainties of their treatments if they are to receive care that is aligned with their priorities and needs.

The presentation and interpretation of information on drug harms has been well researched.^44^ ^45^ ^46^ ^47^ ^48^ How the benefits of new medicines, including any uncertainties surrounding those benefits, are communicated is, however, more limited.^15^ ^49^ An earlier study evaluated the extent to which information on drug benefits is communicated in patient information leaflets in the EU.^50^ But that study did not evaluate the quality of reporting in terms of the accuracy or completeness of the information communicated. The question of whether regulated written information reflects critically appraised evidence on the benefits of anticancer drugs, including key clinical uncertainties and evidence gaps, has not been systematically investigated.

We determined the availability of information on the benefits of all new anticancer drugs approved by EMA during 2017-19 in regulated written information sources for patients. We also assessed the availability of information on the benefits of the drugs in the corresponding sources for clinicians because these leaflets—together with written information for patients—may serve as a tool to enhance communication and shared decision making between patients and clinicians.^51^

## Methods

A content analysis was undertaken of the European public assessment reports (EPARs) and associated written product information for all anticancer drugs granted a first EU marketing authorisation in 2017-19. We systematically compared the information on drug benefits reported in written and electronic sources targeting clinicians (summaries of product characteristics), patients (patient information leaflets), and the public (public summaries) with the information available in the scientific discussion section of EPARs.

### Conceptual and analytical approach

#### Defining patient relevant information on drug benefits

Although it is broadly agreed in the literature that patients want high quality information on drug benefits, consensus is lacking on the types of information that are most relevant and useful for patients.^50^ We therefore reviewed primary research and systematic reviews on the role and value of written information for patients, as well as the grey literature, including good practice guidelines, reflection papers, and reports produced by regulatory bodies and special interest groups.

We derived our initial taxonomy of key benefit information from a content analysis and synthesis of two widely used and validated tools for evaluating the quality of written information on treatments,^52^ ^53^ as well as existing UK and EU regulatory guidance.^4^ ^21^ In addition, we drew on the framework used in a previous study to evaluate information on drug benefits in patient information leaflets.^50^ Our synthesis of these sources gave us six broad categories of information. To verify the relevance and usefulness of these initial categories for patients, we checked them against findings from empirical studies of patients’ treatment information needs and commonly asked questions (see table 1).^3^ ^8^ ^9^ ^10^ ^14^ ^15^ ^16^ ^19^ ^20^ ^22^ ^49^ ^54^ ^56^ ^57^ ^58^ ^59^ ^60^

**Table 1:** Initial taxonomy of key benefit information for patients and consumers

Key types of benefit information	DISCERN^52^	IPDAS^53^	MHRA 2005^21^	EMA 2006^4^	Dickinson et al^50^	Questions commonly asked by patients
What and who the drug is for (indication)	No	No	Yes	Yes	Yes	What disease is this drug used to treat? Is this the right treatment for me?^14^ ^19^ ^54^ ^55^
How the drug works	Yes	No	No	Yes	Yes	How does this drug work?^8^ ^15^ ^22^ ^49^
Goal of treatment	No	No	Yes	Yes	Yes	What is the purpose of treatment—is it to cure disease, prevent disease, prevent the worsening of disease, or to relieve symptoms?^3^ ^9^ ^10^ ^14^ ^19^ ^56^-^58^
Type and source of evidence for drug benefits	Yes	Yes	No	Yes	No	How was the drug studied and how confident can I be in the evidence?^3^ ^9^ ^20^ ^56^
Drug benefits shown	Yes	Yes	No	Yes	Yes	What are the benefits of treatment?^3^ ^9^ ^10^ ^15^ ^16^ ^19^ ^22^ ^55^-^57^
Patient relevant outcomes	No	Yes	No	No	Yes	How will the drug affect the way I feel or function, or how long I survive?^10^ ^20^
Onset and duration	No	Yes	Yes	No	Yes	How long before the medicine will have an effect and how long will the effect last?^10^ ^19^
Magnitude	No	No	No	No	Yes	How large are the benefits?^3^ ^10^
Likelihood	No	Yes	No	No	Yes	How likely are the benefits?^3^ ^9^ ^10^ ^19^ ^20^ ^56^
Uncertainties and gaps in the evidence	Yes	Yes	No	No	Yes	How certain is the available evidence? Are there any important gaps in the evidence?^3^ ^9^ ^20^ ^56^ Does the evidence apply to me?^14^ ^19^ ^55^

EMA=European Medicines Agency; IPDAS=International Patient Decision Aids Standards; MHRA=Medicines and Healthcare products Regulatory Agency.

Our review of this literature confirmed that patients want to know what condition the drug is used to treat, whether it is the right drug for them, and the purpose of treatment. They also want to know how the drug was studied and how confident they can be in the evidence supporting its use, the types of benefit they might expect for patient relevant outcomes (morbidity, quality of life, and mortality), and the likelihood of experiencing those benefits. In addition, patients want information about any evidence gaps or uncertainties related to drug benefits (see table 1).

#### Development of the coding schedule

**Fig 1:**
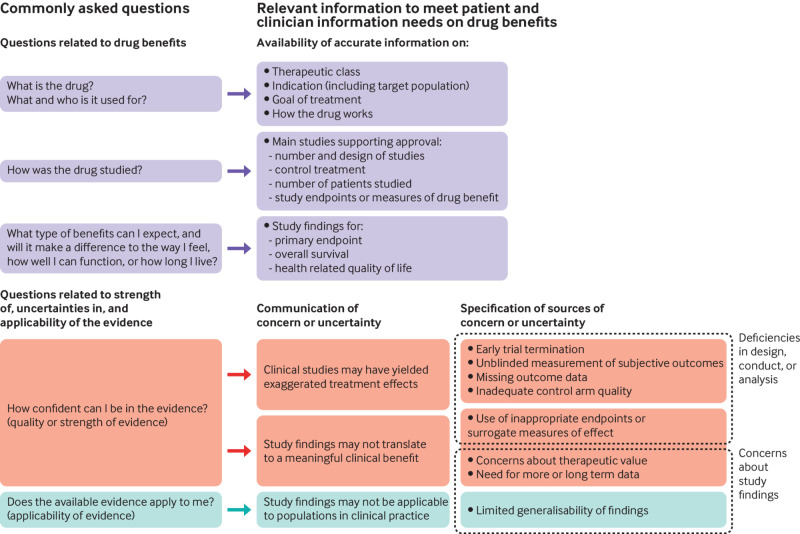
Analytical framework for evaluating the quality of written information on benefits and related uncertainties of prescription drugs

To operationalise these broad categories of information and to develop an evidence informed framework (see fig 1) for data extraction and analysis, we developed predefined coding categories for data extraction that corresponded to patients’ commonly asked questions about drug benefits and related uncertainties. See the supplementary methods for an outline of our coding strategy.

Our coding scheme for evaluating the strength and applicability of the available evidence on drug benefits was informed by empirically documented threats to internal and external validity of clinical study findings. These related to specific aspects of the design, conduct, analysis, and reporting of studies that have been shown to undermine the reliability or interpretation of results for benefit,^61^ ^62^ ^63^ ^64^ or that create uncertainty about the relevance of those results for clinical practice.^65^ ^66^ ^67^ ^68^

In addition, we included a separate category—the need for additional or longer term data—to capture cases where EMA assessors explicitly noted the need for additional data to address evidence gaps and uncertainties related to a drug’s efficacy. For example, the need for comparative data for drugs approved on the basis of single arm trials, or the need for larger sample sizes or longer follow-up to confirm the magnitude or duration of effect when results were based on a small number of patients or interim analyses.

### Analysis

Identification of the study sample and initial analysis of the EPARs and summaries of product characteristics were undertaken by one researcher (JL) and checked by three researchers (CD, HN, and AP). CD and JL extracted data and analysed the public summaries and patient information leaflets, verified by HN. Final analysis of the data was undertaken by CD and checked by HN. Disagreements over the coding of specific items were resolved in the first instance by discussion among JL, AP, CD, HN, and MS-K, then by discussion among all collaborators. Consensus was reached in all cases.

We searched the publicly available EMA database to identify all anticancer drugs with a first marketing authorisation in the EU between 1 January 2017 and 31 December 2019. Consistent with the approach used in a previous study,^34^ we used the Anatomical Therapeutic Chemical Classification codes L01-L04 to retrieve the list of antineoplastic and immunomodulating agents approved within the study period. We cross checked this list against the haematology and haemostaseology section and the cancer section in the *Human Medicines Highlights* reports, which EMA publishes annually to highlight new drugs recommended for approval. We excluded generic, biosimilar, and hybrid products, and treatments for supportive care.

Textual and numerical data pertinent to the analytical categories in our coding scheme were extracted from all documents and entered into a data analysis workbook. Data were analysed at the indication level.

Our initial coding scheme included the category for goal of treatment—a category to record whether the drug was intended to cure, prevent, or alleviate disease. As the goal of treatment was rarely explicitly stated in EPARs, we had to infer whether the treatment was intended to be curative or non-curative (palliative). These inferences (which could be based on the inclusion criteria for the pivotal studies, the approved indication and target population, or general statements in EPARs about the condition and stage of disease for which the treatment was intended) were sometimes difficult to make. Unclear inferences were flagged for discussion and resolved in consultation with an oncologist (AP).

EPARs were used to identify the main clinical studies supporting EMA decisions. We extracted data on drug benefits from EPARs according to the primary study outcomes, as well as information on survival or quality of life benefits when available. Our determination that a statistically significant survival or quality of life benefit had been shown was based on reported hazard ratios, confidence intervals, and P values.

We determined whether there was uncertainty about evidence supporting drug efficacy if EPARs contained an explicit or implicit acknowledgement by EMA assessors of unresolved shortcomings, concerns, or questions corresponding to the predefined list of uncertainty sources as set out in our analytical framework. This included instances where concerns were expressed by scientific advisors consulted during the assessment process. We concluded that EMA assessors had expressed concerns about the therapeutic value of a drug if they questioned the accuracy of estimates of drug efficacy or whether demonstrated benefits were clinically relevant for patients.

For each drug indication in our sample, we systematically compared data extracted from summaries of product characteristics, public summaries, and patient information leaflets with data extracted from EPARs. We judged the information in summaries of product characteristics, patient information leaflets, and public summaries to be accurate if it consistently and comprehensively reflected EMA’s scientific assessment of the clinical evidence. We judged written product information to be inaccurate if it appeared to omit, or only partially report, pertinent information in a way that might lead patients and clinicians to overestimate or underestimate the benefits of a drug or to have unwarranted confidence in the evidence.

We calculated the proportion of summaries of product characteristics, patient information leaflets, and public summaries that communicated relevant and accurate information on drug benefits and uncertainties about benefit.

### Patient and public involvement

Input from individuals with lived experience of cancer treatment has been pivotal in setting out the design and analytical strategy of this study. Our analytical strategy was informed by a literature review of empirical studies of patients’ treatment information needs and commonly asked questions. One member of the author team (HS) has substantial experience in patient advocacy and public involvement roles. We also discussed the relevance and usefulness of our taxonomy of key benefit information with a patient leader from Just Treatment, a UK based patient advocacy organisation.

## Results

### Anticancer drug approvals and sample characteristics

**Fig 2:**
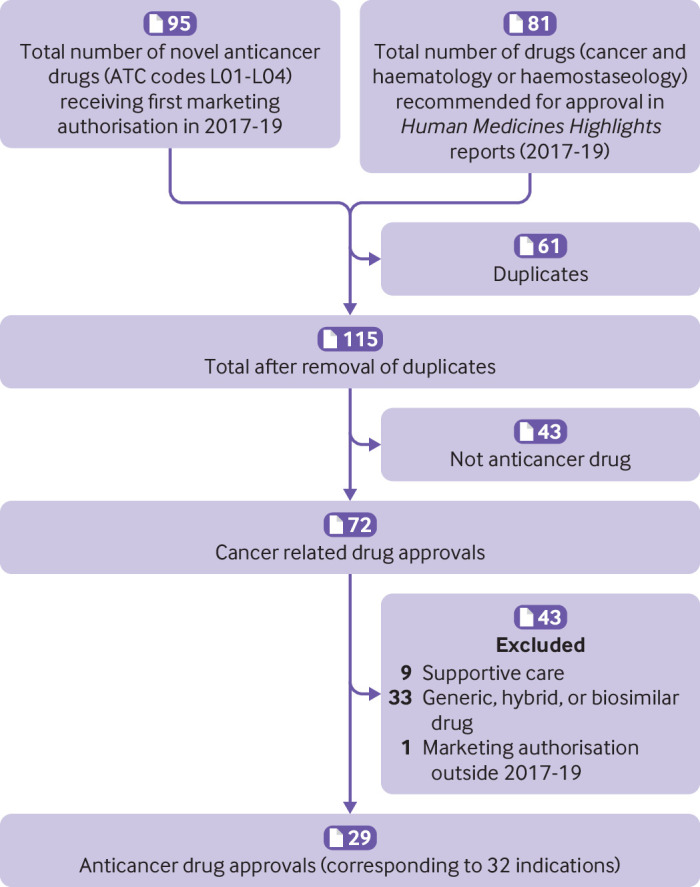
Identification and selection of anticancer drugs receiving a first marketing authorisation during 2017-19. ATC=Anatomical Therapeutic Chemical Classification

The study sample included 29 anticancer drugs that had received a first marketing authorisation between 2017 and 2019 (fig 2). Three were approved for more than one indication, resulting in 29 drugs approved for 32 cancer indications and supported by 37 main studies.

Of the 32 approved indications, 22 (69%) were for the treatment of solid tumours, one was for solid tumours that were histology agnostic, 10 (31%) were for the treatment of haematological malignancies, four (13%) were for early stage disease, and 28 (88%) were for the treatment of advanced or metastatic disease (see supplementary table 1). Based on available information in EPARs, we determined there were four (12%) indications where the goal of treatment appeared to be curative. For the other 28 (88%) indications the goal of treatment was non-curative.

Supplementary table 1 provides further information on the regulatory and treatment characteristics of the sample. Supplementary table 2 provides information on the characteristics of the main studies supporting approval.

### Drug benefits and related uncertainties according to EPARs

#### Evidence of survival or quality of life benefits

Nine out of the 32 (28%) indications showed benefits on patient relevant outcomes of survival or quality of life at the time of EMA approval. The remaining 23 (72%) indications lacked evidence that the drug extended survival or improved quality of life. These drugs were approved on the basis of a surrogate endpoint such as progression-free survival or tumour response.

#### Uncertainties in evidence base for drug benefit

EMA raised concerns about deficiencies in the design, conduct, analysis, or findings of the main studies for nearly all the drugs in our sample. For 31 (97%) indications, EMA questioned whether uncertainties in at least one of the domains included in our analytical framework undermined the reliability or clinical relevance of evidence on drug benefits. EMA raised concerns about at least three and up to six domains for 21 (66%) indications. Of these 21 indications, 11 were granted conditional marketing authorisation or orphan status, or both. For a quarter of indications (8/32), the nature and degree of uncertainty was such that EMA assessors were unable to reach a consensus on whether the benefits of the drug had been shown to outweigh the risks, with a few recommending the drug not be approved.

### Availability of relevant and accurate information in documents

**Fig 3:**
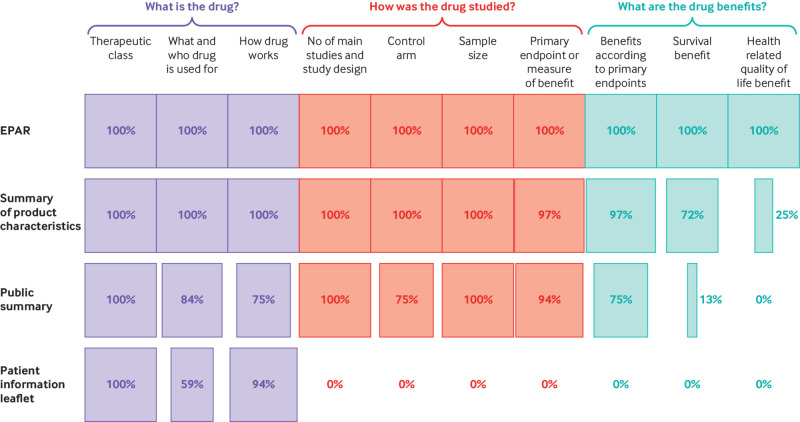
Communication of information about a drug, how it was studied, and evidence of benefit. EPAR=European public assessment report

Figure 3 shows the proportion of summaries of product characteristics, public summaries, and patient information leaflets that reported accurate and complete information about a drug and approved indication, how the drug was studied, and the benefits shown, compared with information reported in EPARs. Compared with other sources of written prescription drug information, patient information leaflets had the lowest rate of reporting across all categories of information. These leaflets did not contain any information on how the drug was studied and its benefits.

#### Communicating general information about a drug

General information about a drug (including information about the approved indication and how the drug works) was reported across most of the documents (fig 3). However, five (16%) public summaries and 13 (41%) patient information leaflets failed to report all relevant information about the approved indication and target patient population (what and for whom the drug is used). Examples of missing information included restrictions to the scope of the indication based on mutational status of patients’ cancers, disease stage, or the availability of alternative treatment options; the approved combination treatment; and how treatments should be sequenced.

#### Communicating information about how a drug was studied

Nearly all summaries of product characteristics (31/32, 97%) communicated accurate and full information about the number and design of the main studies, the control arm (if any), study sample size, and primary measures of drug benefit. The one exception was the summary of product characteristics for ropeginterferon alfa-2b (Besremi), which failed to accurately report the prespecified primary study endpoint (table 2).

**Table 2:** Cases of inaccurate and potentially misleading reporting in summaries of product characteristics and public summaries

Drug and indication	Source and type of inaccurate reporting
Ropeginterferon alfa-2b (Besremi) for the treatment of adults with polycythaemia vera without symptomatic splenomegaly	EPAR mentions that the main study failed to demonstrate the efficacy of ropeginterferon alfa-2b based on the study’s prespecified primary efficacy endpoint. The primary endpoint was changed post hoc. Efficacy could only be demonstrated against one of the study’s secondary endpoints, and only by widening (post hoc) the non-inferiority margin from −10% to −20%. Neither the negative study results, nor the post hoc widening of the inferiority margin for the redefined (post hoc) study endpoint were reported in the summary of product characteristics or the public summary
Atezolizumab (Tecentriq) for the treatment of adults with locally advanced or metastatic urothelial carcinoma after previous platinum-containing chemotherapy or who are considered ineligible for cisplatin	EPAR mentions that the main study for atezolizumab, which was powered to evaluate overall survival as a primary endpoint, failed to demonstrate any survival benefit. Instead of reporting the results as negative, the public summary states that: “patients . . . given Tecentriq lived slightly longer (8.6 months) than patients given chemotherapy (8 months) although the difference could be due to chance”
Rucaparib (Rubraca) for the treatment of adults with platinum sensitive, relapsed or progressive, BRCA mutated, high grade epithelial ovarian, fallopian tube, or primary peritoneal cancer	Public summary reports a tumour response rate of 65% for a subset of participants with platinum sensitive disease. This subgroup was defined post hoc. The response rate for the primary efficacy population (54%) was not reported
Tisaglenlecleucel (Kymriah) for the treatment of adults with relapsed or refractory diffuse large B cell lymphoma after two or more lines of systemic treatment	Main study was a single arm trial, which is not reported in the public summary. In reporting the results of the main study, the public summary states that results “were comparable to those from studies of patients receiving standard cancer treatments.” The statement is potentially misleading because patients could interpret this to mean that the drug demonstrated comparable efficacy to alternative treatment options
Tisaglenlecleucel (Kymriah) for the treatment of children and young adults up to 25 years of age with B cell acute lymphoblastic leukaemia that is refractory, who are in relapse post-transplant, or who are in second or later relapse	Main study was a single arm trial, which is not reported in the public summary. The public summary states that the result “was better than results seen with the cancer medicines clofarabine, blinatumomab or a combination of clofarabine, cyclophosphamide and etoposide.” The statement is potentially misleading because patients could interpret this to mean that the drug demonstrated superior efficacy to alternative treatment options
Larotrectinib (Vitrakvi) for the treatment of tumours that display a neurotrophic tyrosine receptor kinase gene fusion, who have a disease that is locally advanced, metastatic, or where surgical resection is likely to result in severe morbidity; and who have no satisfactory treatment options	Public summary suggests that the drug will improve patients’ quality of life when no benefits were demonstrated with respect to any of the quality-of-life measures evaluated in the main study

EPAR=European public assessment report.

The proportion of public summaries that contained accurate information on how drugs were studied was generally high (75-100%), depending on the category of information reported. The primary measures of drug benefit were accurately reported in 94% of public summaries. Two public summaries failed to accurately report study endpoints (table 2).

A quarter of the public summaries in the sample (8/32) failed to accurately report the study design. In all cases this was because the document did not report that at least one of the main studies supporting approval lacked any comparator treatment. Of the 14 indications where at least one of the main studies was a single arm trial or lacked a comparator, this was accurately communicated in six cases. Supplementary table 3 illustrates how the study design was communicated for selected drugs.

None of the patient information leaflets reported relevant information about the evidence for drug benefits or how the drugs had been studied (number and design of the main studies, nature of the control arm (if any), study sample size, or primary measures of drug benefit).

#### Communicating information about drug benefits

Thirty one (97%) summaries of product characteristics and 24 (75%) public summaries contained complete information about the benefits patients might expect from a drug based on the primary study endpoints. Most of the drugs in our sample (23/32, 72%) had not been shown to extend survival or improve quality of life. Instead, these drugs were approved based on surrogate measures of drug efficacy. Supplementary table 4 provides examples of the ways in which drug benefits based on the two most commonly evaluated surrogate endpoints were described in public summaries.

One (3%) summary of product characteristics and seven (21%) public summaries reported drug benefits in a way that was inconsistent with information in the EPAR and potentially misleading (table 2). An example is the public summary for larotrectinib (Vitrakvi) for the treatment of patients with advanced or metastatic solid tumours that display a neurotrophic tyrosine receptor kinase gene fusion. Although the document accurately reported quantitative results for the proportion of patients with a reduction in the size of their tumours (the overall response rate), the section describing why the drug was approved contained a statement implying that tumour response would translate into a quality-of-life benefit for patients (“In addition, the short time taken to shrink the tumours is important in relieving patients’ symptoms”). However, larotrectinib showed no benefit for any of the quality-of-life measures included in the study.

The presence or absence of evidence that a drug extended life was reported in 23 (72%) summaries of product characteristics and four (13%) public summaries. In all four cases where public summaries reported whether or not a drug had been shown to prolong survival, a statistically significant survival benefit had been shown. For the remaining 28 indications, public summaries either failed to report evidence of a statistically significant survival benefit (three cases) or failed to report a lack of evidence that the drug prolonged patients’ lives (25 cases). The presence or absence of evidence that a drug improved quality of life was reported in eight (25%) summaries of product characteristics and in no (0%) public summaries. Among the 24 summaries of product characteristics that did not include information on whether a drug had been shown to improve quality of life were two cases where a quality-of-life benefit had been demonstrated and 22 cases where evidence that the drug improved patients’ quality of life was lacking.

None of the patient information leaflets in our sample communicated any information about the types of drug benefits that patients might expect based on study findings (fig 3). In contrast, nearly all patient information leaflets (94%) described the drug’s mechanism of action (how a drug works in the body). Supplementary box 1 illustrates six examples of statements typically contained in patient information leaflets, including phrases such as: “allow[s] the immune system to attack the tumour cells,” “helps your immune system to fight your cancer,” “caus[es] the death of cancer cells,” “destroy[s] cancer cells,” and “trigger[s] the death of cancer cells.” None of the drugs included in supplementary box 1 had shown a survival or quality-of-life benefit, and all were indicated for the treatment of metastatic or late stage disease.

#### Communicating concerns and uncertainties about drug benefits

**Fig 4:**
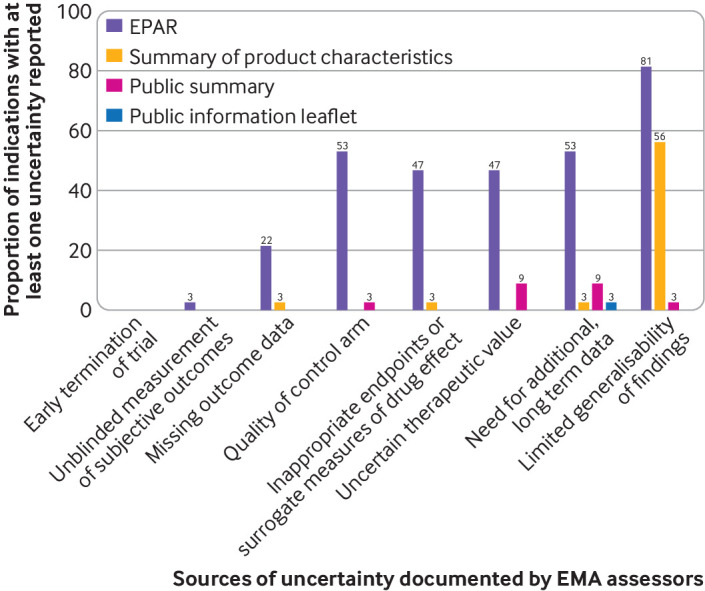
Communication of EMA assessors’ concerns about study methods and findings. EMA=European Medicines Agency; EPAR=European public assessment report

Figure 4 shows the percentage of EPARs in our sample where a concern was raised about each of the uncertainty domains included in our analytical framework, and the proportion of summaries of product characteristics, public summaries, and patient information leaflets that reported those concerns. Regulators’ concerns about the reliability or clinical relevance of evidence on drug benefits were rarely communicated to clinicians or patients—with the partial exception of concerns around the non-generalisability of findings—even when EMA’s scientific assessors were unable to reach a consensus on whether the drug should be approved. For example, EMA assessors could not agree on the approval of atezolizumab for the treatment of locally advanced or metastatic urothelial carcinoma owing to “substantial uncertainties regarding the efficacy of [the drug]” (see supplementary table 5). Approval of first line use of atezolizumab in this indication was based on one single arm trial in which response rates were acknowledged to be inferior when indirectly compared with an historical control. Approval in the second line setting was based on a randomised controlled trial in which atezolizumab failed to meet the primary endpoint of improved overall survival compared with chemotherapy. Regulatory concerns over the clinical efficacy of atezolizumab were not communicated in the written information for clinicians or patients. Instead, the public information leaflet stated: “Tecentriq helps your immune system to fight your cancer.”

Supplementary table 5 provides further illustrative examples of drugs where EMA assessors and scientific advisors raised multiple concerns in EPARs, and compares comments with the information communicated about drug benefits (or in the case of patient information leaflets, drug mechanisms of action) and related uncertainties in the corresponding public summaries and patient information leaflets.

## Discussion

In this study we examined the extent to which information on the benefits of drugs and related uncertainties was communicated in regulated prescription drug information in Europe for 32 anticancer drug indications with a first marketing authorisation between 2017 and 2019.

Both patient facing and public facing sources on drug information often lacked relevance: information on drug benefits was not reported in any patient leaflets, whereas other, potentially less relevant information for patients (ie, the biological mechanism of action) was consistently included. We also found instances where the reporting of a study design and study findings was inconsistent with the information reported in EPARs and potentially misleading. Finally, written information was often not comprehensive: the availability or absence of evidence on patient important outcomes (including overall survival and quality of life) was rarely explicitly reported, and uncertainties in the evidence as identified by the regulatory assessors were mostly omitted from written information sources.

Our findings highlight important shortcomings in written information for prescription drugs in Europe. Studies confirm that patients want information about the benefits of drugs as well as the risks.^3^ ^9^ ^10^ ^14^ ^15^ ^16^ ^19^ ^20^ ^22^ ^54^ ^55^ ^56^ ^57^ ^58^ ^60^ Despite this need, basic information on benefit in multiple categories was not always communicated in regulated product information for the drugs in our sample. For example, important information about the goal of treatment—whether a drug is intended to prevent or cure disease or to be palliative—had to be inferred from EMA’s scientific assessment reports in most cases and was rarely explicitly stated in the written information for patients or clinicians.

Studies also show that patients want information on the strength of evidence and any relevant clinical uncertainties or evidence gaps.^3^ ^9^ ^14^ ^19^ ^20^ ^55^ ^56^ ^60^ But reporting of information to address these concerns was extremely limited. The existence of critical knowledge gaps for outcomes most important to patients was rarely made explicit in information products for patients and the public—and it cannot be assumed that this information will be communicated to patients by clinicians. Consistent with previous research, we found low rates of reporting of quality-of-life outcomes in the summary of product characteristics for healthcare professionals.^69^

In line with previous studies,^32^ ^34^ ^36^ we found that the evidence supporting approval of many new anticancer drugs in Europe is prone to major methodological limitations. European regulators often raised concerns about deficiencies in the design, conduct, analysis, or findings of studies to support marketing authorisation. Regulators’ concerns about treatment efficacy were rarely communicated to clinicians or patients. While a relatively high proportion of summaries of product characteristics and public summaries (but no patient information leaflets) included technically accurate information about how drugs had been studied, communication was not explicit in the public summaries about the relevance of different study designs and endpoints for the strength, reliability, and clinical relevance of the evidence. Unless patients have some previous understanding of the implications of different research methodologies, it is unlikely that the information currently communicated addresses patients’ concerns about evidence quality.

### Policy implications

Our study highlights the need to improve the communication of drug benefits and related uncertainties in regulated prescription drug information in Europe. The provision of high quality information on drug benefits and related uncertainties is especially important for patients with time limiting conditions such as advanced cancer. Regulatory approval of an apparently effective new treatment is an important source of hope for patients with cancer. A major body of research shows that many patients with cancer, particularly those with advanced disease, misunderstand the purpose of drug treatment and overestimate the benefits.^38^ ^39^ ^40^ ^41^ ^70^ ^71^ ^72^ ^73^ ^74^ ^75^ ^76^ ^77^ When patients have unrealistic expectations of the benefits from treatment and misplaced confidence in the strength of study findings underlying approvals, informed decision making is undermined.

Despite the commitment of medicines regulators to shared decision making and person centred care,^78^ current regulated sources of prescription information in Europe do not allow patients to distinguish between new anticancer drugs that offer clinically meaningful benefits compared with those with considerable uncertainty about effects. In fact, by failing to communicate evidence gaps and uncertainties, current product information risks exacerbating and perpetuating common misconceptions about new drugs. Recent research suggests that in the absence of explicit information about the strength of the evidence underpinning recommended treatments and interventions, people assume the evidence is of high quality.^79^ And without a clear statement in the product information for new anticancer drugs that surrogate endpoints such as progression-free survival and tumour response do not reliably predict either patient survival or improved quality of life,^80^ ^81^ ^82^ ^83^ it is unclear how patients will interpret standard descriptions of these endpoints in EMA’s public summaries. The small body of research investigating this topic suggests the terminology typically used in public summaries (“increasing the time patients live without their disease getting worse,” “the cancer shrank or was eliminated,” “the disease responded to treatment”) will lead to confusion about the medicine’s purpose and the types of benefits patients can expect from a drug.^31^ ^37^ ^42^ ^84^ ^85^

Patients may have access to regulated information about prescription drugs only after a treatment decision has already been made. Even in such cases, better information on medicines can address patients’ evolving information needs during treatment, and help inform decisions about if and when to discontinue treatment.^19^ Patients may also seek information not only on their prescribed medicines but also on other available treatment options. Better sources of regulated prescription drug information can have spill-over effects for broader information about medicines; many stakeholders in the health system (including patient organisations, charities, and health technology assessment bodies) rely on regulated information sources when communicating information about new drugs.

We recognise that regulators cannot compel companies to include information on drug benefits and evidence about uncertainties of benefits in patient package leaflets, because current UK and EU legislation does not mandate this information. However, mechanism of action statements may be misunderstood as statements of effectiveness, especially in the absence of qualifiers, reporting on uncertainty, or quantitative benefits information. This may be at odds with legal requirements for information in patient leaflets to be accurate, non-misleading, and non-promotional. In addition, regulators could concentrate efforts on ensuring that relevant, accurate, and useful content are available in online lay summaries for the public. Although public summaries are produced in line with regulated templates, these templates could be amended without the need for legislative change. Information on benefit could be presented as part of an anticancer drug specific “drug facts box” and made available online to inform shared decision making in clinical practice.^86^ ^87^ Further research may be needed to determine how best to communicate information on evidence uncertainties and how to strike the right balance between too much and too little information.

### Limitations of this study

Our study has limitations. Firstly, our initial taxonomy of key benefit information, although based on an extensive review of the academic and grey literature, may not have captured all information about drug benefits and uncertainties that might be relevant and useful for patients.

Secondly, our coding scheme could not include every category of information that we identified from the literature. For example, we did not include information on the average time before patients could expect to experience benefits, because this information was not always available in EPARs.

Thirdly, we have not captured every aspect of a trial’s design, conduct, or analysis that could affect the strength, reliability, or relevance of the evidence. For example, we did not examine the extent to which the population studied in the main trials was the same as the population for which the drug was ultimately approved (although discrepancies may sometimes have been captured in regulatory concerns about generalisability or concerns about the therapeutic value of the drug in certain patient populations).

Fourthly, in relying on EMA assessments for our determination of evidence uncertainties rather than some objective measure or instrument (such as the Cochrane risk of bias tool), we may have underestimated the extent to which problematic study methods undermined results. For example, EMA assessors flagged concerns about the effects of missing outcome data and the unblinded measurement of study outcomes on the reliability of findings in a smaller proportion of EPARs relative to the frequency with which EMA assessors expressed concerns in relation to the other uncertainty domains. In contrast, a previous study of EMA anticancer drug approvals using the Cochrane risk of bias tool found that incomplete outcome data and measurement of the outcome were the primary domains responsible for high risk of bias judgments.^34^

Finally, our analysis includes only new anticancer drugs approved between 2017 and 2019, and it is not clear whether our findings can be generalised to other disease areas. However, anticancer drugs now comprise the single largest category of new drug approvals in Europe.

### Conclusion

Regulated information sources for anticancer drugs in Europe fail to address the information needs of patients. This study identified important shortcomings in the communication of information on drug benefits and related uncertainties in regulated sources. If patients lack access to such information, clinical decisions may not align with their preferences and needs.

**What is already known on this topic**

To receive and participate in medical care, patients need high quality information about the benefits and risks of prescription drugs
Earlier studies investigated how information on drug risks and adverse effects is communicated to patients, but research on communication of drug benefits is limited

**What this study adds**

The benefits of anticancer drugs are rarely well communicated to patients in regulated information sources for prescription drugs in Europe
Regulators’ concerns about the reliability and interpretation of evidence for the benefits of anticancer drugs are rarely available in regulated information sources aimed at clinicians or patients

## Data Availability

No additional data available.

## Web extra

Extra material supplied by authors

Supplementary information: Additional methods, tables 1-5, and boxes

## Notes

Contributors: CD and HN conceived and designed the study and developed the analytical framework. CD, JL, MS-K, and HN collected data. CD independently verified all collected data, undertook the primary analysis, and drafted the manuscript. All authors contributed to subsequent iterations. All authors provided critical input on the manuscript and approved the final version for publication. CD is guarantor. The corresponding author attests that all listed authors meet authorship criteria and that no others meeting the criteria have been omitted.

The lead author (CD) affirms that the manuscript is an honest, accurate, and transparent account of the study being reported; that no important aspects of the study have been omitted; and that any discrepancies from the study as planned have been explained.

Dissemination to participants and related patient and public communities: We have secured funding from King’s College London’s Economic and Social Research Council (ESRC) Impact Acceleration Account (principal investigator: CD, co-investigator: HN) to continue our engagement with patient and consumer health groups and other key stakeholders, including regulators, charities, and health information organisations after publication of results.

Provenance and peer review: Not commissioned; externally peer reviewed.
