# Supplementary material for: Communication of anticancer drug benefits and related uncertainties to patients and clinicians: document analysis of regulated information on prescription drugs in Europe
Source: BMJ. 2023 Mar 29;380:e073711. doi: 10.1136/bmj-2022-073711 (PMC10053600; doi:10.1136/bmj-2022-073711)
Supplement: Supplementary file 1 — Supplementary information: Additional methods, tables 1-5, and boxes [file davc073711.ww.pdf]

Communication of cancer drug benefits and uncertainties to patients and clinicians: Document analysis of regulated prescription drug information in Europe

Courtney Davis, Anita K. Wagner, Maximilian Salcher-Konrad, Henry Scowcroft, Barbara Mintzes, Adrian M.J. Pokorny, Jian Lew, Huseyin Naci

Appendices

Appendix methods

Detailed coding scheme and strategy

Availability of information on drug benefits
In our coding scheme for evaluating whether written product information addressed patients' general questions about the drug and the benefits they might expect, we examined the availability of information on the following items:

**Broad domain**	**Specific categories of information**
What the drug is	Type of medicine and name of the active substance
What and who the drug is used for	Indication and target population, including:<br>• type of cancer;<br>• stage of disease;<br>• any special characteristics of the disease or patient (e.g., specific tumour markers, high/low risk disease, subgroups not eligible for treatment);<br>• line of therapy;<br>• sequencing of therapy;<br>• specification of approved combination treatment
How the drug works	Drug's mechanism of action in the body
The purpose of drug treatment	Whether the drug is intended to cure disease, reduce the risk of disease, prevent the worsening of disease, or palliate disease symptoms
How was the drug studied	Number of main studies supporting approval and key aspects of study design, including<br>• whether studies were: randomised controlled trials, non-comparative trials (including dose-comparator) randomised trials, single arm studies, or some other type of study design;<br>• what treatment the experimental drug was compared to, if any;<br>• how many patients participated the studies;<br>• how the studies evaluated drug efficacy (the study endpoints)
Benefits shown	Study findings (included the nature and magnitude of any demonstrated benefits) with respect to:<br>• pre-specified primary study endpoint(s);<br>• overall survival;<br>• health-related quality of life

Our coding scheme did not include all categories of information listed in our initial taxonomy (Table 1).

Our initial taxonomy also included the category: 'onset and duration of drug benefit' – that is, how long a patient should expect to wait before experiencing drug benefits, and how long those benefits would be expected to last. However, this information was not routinely available for all drugs in the sample so we did not include this category in the final coding scheme.

<u>Availability of information on benefit uncertainties</u>
In our coding scheme for evaluating whether written product information addressed patients' concerns about the strength, clinical relevance and applicability of the available evidence with respect to drug benefits, we examined the availability of information on the following items:

**Broad domain**	**Specific categories of information**
Strength/ quality of evidence<br>(threats to internal validity)	Regulatory concerns that studies may have yielded exaggerated treatment effects due to deficiencies in design, conduct and/or analysis, specifically:<br>• whether studies were terminated early;<br>• whether measurement of subjective study outcomes was unblinded;<br>• whether studies suffered from missing outcome data;<br>• whether studies lacked a control arm, or lacked an appropriate control;
Clinical relevance of evidence	Regulatory concerns that study findings may not translate to a meaningful clinical benefit because:<br>• the magnitude of the effect was small/not clinically relevant;<br>• studies included inappropriate endpoints or unvalidated, surrogate measures of drug efficacy
Applicability of evidence<br>(threats to external validity	Regulatory concerns that study findings may not be generalisable to specific populations in clinical practice

We also collected information on the regulatory and therapeutic characteristics of the sample, including whether drugs were granted a conditional marketing authorization or any special designation – for example, orphan designation, 'advanced therapy medicinal product' classification, or designation as a priority medicine under the EMA's PRIME scheme.

For drugs granted a conditional marketing authorisation, all SmPCs and Public Summaries should contain a statement acknowledging the conditional status of the market authorisation and making some reference to the additional postmarketing studies required for full approval. However, where this statement did not specify the nature of

the evidence gap or uncertainties that postmarketing studies were meant to address, we did not consider the statement to have reported relevant uncertainties.

Detailed information on data sources

European Public Assessment Reports (EPARs) related to the initial marketing authorisation for all drugs in our sample were retrieved from the 'Initial marketing-authorisation documents' section of EMA's database of human medicines (https://www.ema.europa.eu/medicines).

Because the Summary of Product Characteristics (SmPCs), Public Summaries and Patient Information Leaflets (PILs) in EMA's database are updated following approval of line extensions and as new information becomes available, original SmPCs and PILs (matching the versions available at market entry) were obtained from the European Commission's Union Register of Medicinal Products for Human Use (https://ec.europa.eu/health/documents/community-register/html/), which contains a chronological listing of current and archived versions of the product information approved since product launch. There is no similar archive for Public Summaries. However, we were able to identify and extract the information in the Public Summaries relevant to each drugs' initial marketing authorisation.

Appendices - Results

Appendix Table 1. Regulatory and therapeutic characteristics of the sample according to the EPARs

Characteristics	N = 32 (%)
**Year of Approval**	
2017	12 (37.5)
2018	12 (37.5)
2019	8 (25.0)
**Regulatory Approval Characteristics**	
Orphan Designation	13 (40.6)
Conditional Marketing Authorisation	6 (18.8)
Approval Under Exceptional Circumstances	0 (0.0)
Advanced Therapy Medicinal Product Classification	3 (9.4)
Priority Medicine Classification (PRIME)	2 (6.3)
Regular Approval	26 (81.3)
**Cancer Type**	
Solid tumours	22 (68.8)
Haematological malignancies	10 (31.3)
**Disease stage [a]**	
Early or newly diagnosed	4 (12.5)
Advanced, metastatic, relapsed or refractory	28 (87.5)
**Route of Administration**	
Oral	18 (56.3)
Injectable	14 (43.8)

[a] Categories are based on the terminology used in the EMA-approved indications for these drugs. 'Early' and 'newly diagnosed' are not equivalent and thus these categories are arguably ambiguous; however, for simplicity and clarity, we have relied on the EMA descriptions as stated.

Appendix Table 2. Characteristics of main studies supporting approval according to the EPARs

**Characteristics**	**N of studies = 37 (%)** [a]
**Study design**	
Randomised trial	22 (59.5)
Non-comparative randomized trial [b]	2 (5.4)
Single-arm study	13 (35.1)
**Control arm**	
Placebo	8 (21.6)
Active	8 (21.6)
Physician's choice	3 (8.1)
No intervention (active surveillance)	1 (2.7)
Add-on design	2 (5.4)
Dose comparator	1 (2.7)
Other	1 (2.7)
No comparator	13 (35.1)
**Sample size**	
0-99	4 (10.8)
100-499	21 (56.8)
500-999	10 (27.0)
1000-2999	2 (5.4)
**Primary measure(s) of drug efficacy**	
Overall survival	5 (13.5)
Overall survival and progression-free survival (joint)	1 (2.7)
Overall survival and tumour response (joint)	1 (2.7)
Quality of life	0 (0)
Progression-free survival	10 (27)
Tumour response	14 (37.8)
Other	5 (13.5)
None	1 (2.7)
**Overall survival or quality of life included as secondary outcome**	
Overall survival studied as a secondary outcome	27 (73.0)
Quality of life studied as a secondary outcome	29 (78.4)

[a] Denominator corresponds to the total number of studies (n=37) supporting the approval of 32 cancer drug indications during our study period.
[b] These include one dose-comparator study, and one study where a combination of two experimental drugs was compared to one of the experimental drugs alone.

Appendix Table 3. Reporting of study design in Public Summaries and PILs – selected examples

**Drug**	**Indication**	**Information from section 'What benefits of drug X have been shown in studies? in EPAR summaries**	**Information in PILs**
Alecensa (alectinib)	Indicated for the treatment of adult patients with ALK-positive advanced non-small cell lung cancer previously treated with crizotinib	"Two main studies involved a total of 225 patients in whom the disease progressed despite previous treatment with Xalkori (crizotinib). **In both studies Alecensa was not compared with any other treatment or placebo (a dummy treatment).** Response to treatment was assessed using body scans and standardised criteria for solid tumours, with complete response being when the patient had no remaining signs of the cancer."<br><br>**Comment**: lack of control arm clearly reported	***No information***
Libtayo (cemiplimab)	Indicated for the treatment of adult patients with metastatic or locally advanced cutaneous squamous cell carcinoma who are not candidates for curative surgery or curative radiotherapy	"In a main study involving a total of 193 patients, the cancer shrank in around 39% of patients with metastatic disease who received 350 mg Libtayo every 3 weeks for around one year. Among patients with locally advanced disease who received Libtayo every 2 weeks (at a dose of 3 mg/kg bodyweight) for around 2 years, 44% of patients showed shrinkage of their cancer."<br><br>**Comment:** no information on lack of control arm	***No information***
Kymriah (tisangenlecleucel)	Indicated for the treatment	"The main study of Kymriah in B-cell ALL involved 92 children and	***No information***

	of… young adult patients up to 25 years of age with relapsed or refractory B-cell acute lymphoblastic leukaemia	young adults (3–25 years of age) whose cancer had come back after previous treatment or did not respond to treatment. Around 66% of patients had a complete response (which means they had no signs of the cancer left) in the 3 months after treatment. **This was better than results seen with the cancer medicines clofarabine, blinatumomab or a combination of clofarabine, cyclophosphamide and etoposide**. **Twelve months after treatment, the likelihood of survival was 70%.”**<br><br>**Comment:** potentially misleading communication of the study design and results of this single arm trial, which was compared by the sponsor to response rates found with historic controls	

Appendix Table 4. Examples of benefit reporting in Public Summaries when results were based on surrogate endpoints

**Drug**	**Text from Public Summary when study endpoint was progression-free survival**
**Niraparib** (Zejula)	"Patients treated with Zejula lived on average 11.3 months without their disease getting worse compared with 4.7 months in patients treated with placebo (a dummy treatment)... This may allow the next cycle of platinum-based therapy to be delayed."<br><br>***Comment:***<br>• *No explanation of what 'without their disease getting worse' means*<br>• *Explains what this might mean in terms of a clinically relevant outcome*
**Tivozanib** (Fotivda)	"Patients taking Fotivda lived for longer without their disease worsening (12 months) than those given another approved medicine sorafenib (9 months)."<br><br>***Comment:***<br>• *No explanation of what 'without their disease getting worse' means*<br>• *No explanation of what this might mean in terms of a clinically relevant outcome*
**Drug**	**Text from Public Summary when study endpoint was response rate**
**Inotuzumab ozogamicin** (Besponsa)	"The main measure of effectiveness was response to treatment. Patients were considered to have responded if they had no remaining cancerous B cells in their blood and bone marrow after treatment. An analysis of the first 218 patients treated showed that after at least 2 cycles of treatment, 81% (88 out of 109) of patients receiving Besponsa responded to treatment compared with 29% (32 out of 109) of patients receiving other chemotherapy. Patients who responded to treatment could proceed to have a stem cell transplant."<br><br>***Comment:***<br>• *Explains 'response rate' in lay terms*<br>• *Explains what this means in terms of a clinically relevant outcome*
**Alectinib** (Alecensa)	"Response to treatment was assessed using body scans and standardised criteria for solid tumours, with complete response being when the patient had no remaining signs of the cancer. In the first study around 52% of patients given Alecensa (35 out of 67) were considered by the treating doctors to have shown a complete or partial response to the medicine at the time of analysis. In the second study, the complete or partial response rate at the time of analysis was 51% (62 out of 122 patients). Response was maintained for an average of 14.9 months in the first study, and 15.2 months in the second study."<br><br>***Comment:***

	• *Incomplete explanation of response rate. Describes 'complete response' in lay terms, but no explanation of 'partial response'*<br>• *No explanation of what this might mean in terms of a clinically relevant outcome*
**Midostaurin** (Rydapt)	"Overall, using the most stringent, up-to-date criteria, the disease responded to treatment in about 28% of patients (32 of 113). When looked at separately the response rate was highest (60%) in those with aggressive systemic mastocytosis."<br><br>***Comment**:*<br>• *No explanation of what 'responding to treatment' means*<br>• *No explanation of what this means in terms of a clinically relevant outcome*

Appendix Box 1. Communication of mechanism of action in PILs

Alectinib (Alecensa) for ALK-positive advanced NSCLC:

- "Alecensa blocks the action of an enzyme called 'ALK tyrosine kinase'. Abnormal forms of this enzyme (due to fault in the gene that makes it) help encourage cancer cell growth. Alecensa may **slow down or stop the growth of your cancer. It may also help to shrink your cancer**."

Avelumab (Bavencio) for metastatic Merkel cell carcinoma:

- "PD-L1 is found on the surface of MCC cells, and helps protect tumour cells from the immune system (the body's natural defences). Bavencio **binds to PD-L1, and blocks this protective effect, allowing the immune system to attack the tumour cells**."

Lutetium (177Lu) oxodotreotide (Lutathera) for unresectable or metastatic somatostatin receptor positive GEP-NETs:

- "The tumour needs to have somatostatin receptors on the surface of its cells in order for the medicine to be effective. Lutathera **binds with these receptors and emits radioactivity directly into the tumour cells, causing their death**."

Tisagenlecleucel (Kymriah) for relapsed or refractory DLBCL:

- "The T cells are taken from your blood and a new gene is put into the T cells so that they can then find the cells causing your cancer. When Kymriah is infused into your blood, **the modified T cells will find the cancer cells and destroy them**."

Lorlatinib (Lorviqua) for ALK-positive advanced NSCLC:

- "Lorviqua inhibits a type of enzyme called tyrosine kinase and **triggers the death of cancer cells in patients with alterations in genes for ALK**. Lorviqua is only given to patients whose disease is due to an alteration in the gene for ALK tyrosine kinase."

Cemiplimab (Libtayo) for metastatic or locally advanced cutaneous squamous cell carcinoma:

- "LIBTAYO works by **helping your immune system fight your cancer**."

Appendix Table 5: Examples of reporting of drug benefits and benefit uncertainties in Public Summaries and PILs

Drug	Indication	Nature of EMA concerns about study methods and findings as reported in EPAR	Information from sections 'What benefits of drug X have been shown in studies?' and "Why is drug X approved?' in Public Summaries	Information from section 'How does drug X work?' in PILs
**Examples where uncertainties were not accurately reported in the Public Summary or PIL**				
**Tivozanib** (Fotivda)<br><br>Approved 24/08/2017<br><br>Regular marketing authorisation	Treatment of adult patients with advanced renal cell carcinoma and for adult patients who are VEGF and mTOR pathway inhibitor-naïve following disease progression after one prior treatment with cytokine therapy for advanced renal cell carcinoma	Inappropriate comparator<br>Regulators "expressed concern that sunitinib rather than sorafenib was currently considered to be the most active tyrosine kinase inhibitor… in first-line [renal cell carcinoma]" (p. 90).<br><br>Inconsistency in subgroup findings<br>"Internal inconsistency is noted in the large subgroups by geographical region. In the large subgroup of patients from Russia or Ukraine (56% of patients), the hazard ratio for [progression-free survival] is 0.94 (0.70-1.26). No imbalance in prognostic factors was identified as a possible background cause for lack of [progression-free survival] treatment effect in the large Russian/Ukrainian subgroup." (p92)	"A main study of 517 patients with advanced renal cell carcinoma that had either come back or spread to other parts of the body has shown that Fotivda can help stop the disease from getting worse. In this study, patients taking Fotivda lived for longer without their disease worsening (12 months) than those given another approved medicine sorafenib (9 months)."	"Tivozanib reduces the supply of blood to the cancer, which *slows down the growth and spread of cancer cells*. It works by blocking the action of a protein called vascular endothelial growth factor (VEGF). Blocking the action of VEGF prevents the formation of new blood vessels."<br>(Emphasis added)

Drug	Indication	Nature of EMA concerns about study methods and findings as reported in EPAR	Information from sections 'What benefits of drug X have been shown in studies?' and "Why is drug X approved?' in Public Summaries	Information from section 'How does drug X work?' in PILs
		Divergent opinion within EMA's Scientific Committee<br>"In our view, the clinical efficacy of tivozanib has not been sufficiently established. The results of the single pivotal trial are not compelling, and it is also of concern that a large proportion of patients started a new anti-cancer therapy before progression. Considering the open-label design of the single pivotal study, [overall survival] needs to be reassuring (in line with scientific advice and EMA guidance). However, all performed [overall survival] analyses have failed to show any [overall survival] benefit, and thus [overall survival] results do not support the [progression-free survival] results." (p123)		
**Atezolizumab** (Tecentriq)	Treatment of patients with locally advanced	Lack of comparator arm for one of the main studies	"In a study of 429 patients, the cancer shrank or was eliminated after Tecentriq treatment in 23% of patients	"Tecentriq works by attaching to a specific protein in your body

Drug	Indication	Nature of EMA concerns about study methods and findings as reported in EPAR	Information from sections 'What benefits of drug X have been shown in studies?' and "Why is drug X approved?' in Public Summaries	Information from section 'How does drug X work?' in PILs
Approved 20/09/2017<br><br>Regular marketing authorisation	or metastatic urothelial carcinoma (1) after prior platinum-containing therapy (second-line) or (2) who are considered cisplatin ineligible (first-line)	"The non-randomized trial design in this submission is considered a large drawback, since comparisons of time-related endpoints and prognostic characteristics of study populations are associated with uncertainties" (p192).<br><br><u>Small and uncertain treatment effects</u><br>"Although overall response rates of atezolizumab compare less favourably to the best historical comparator of CarboGem (22.7% vs. 36.1%), responses were ongoing in 70% of patients with a median follow-up of 17.2 months (compared to 5.3 months for Carbo/Gem)" (p.196).<br><br><u>Failure to demonstrate survival benefit in second main study</u><br>"Study IMvigor 211 did not demonstrate statistical significance in the primary [overall survival] analysis. [Overall survival] data for atezolizumab were numerically superior to [standard of care]" (p193)	who were not eligible for platinum chemotherapy and in 16% of patients who had previously had platinum chemotherapy."<br><br>"In another study involving 931 patients with urothelial cancer, those given Tecentriq lived slightly longer (8.6 months) than patients given chemotherapy (8 months) although the difference could be due to chance."	called programmed death-ligand 1 (PD-L1). This protein suppresses the body's immune (defense) system, thereby protecting cancer cells from being attacked by the immune cells. *By attaching to the protein, Tecentriq helps your immune system to fight your cancer*."<br>(Emphasis added)

Drug	Indication	Nature of EMA concerns about study methods and findings as reported in EPAR	Information from sections 'What benefits of drug X have been shown in studies?' and "Why is drug X approved?' in Public Summaries	Information from section 'How does drug X work?' in PILs
		Divergent opinion within EMA's Scientific Committee<br>"Current evidence on efficacy and safety in first-line cisplatin-ineligible patients only comprises a single arm study… where the response rate may be considered low. Comparison is made indirectly to CarboGem and does not allow concluding on an advantage for Tecentriq over CarboGem due to limitations related to indirect cross-trial comparison. Furthermore, efficacy outcomes of [overall response rate] and [progression-free survival] are considered inferior to CarboGem…The lack of direct comparative efficacy data with first line agents precludes a determination of the extent of any potential "loss of chance". In conclusion, the uncertainties in current data outweigh the favourable safety profile… With regard to the second-line setting, ... [t]he design of study IMvigor 210 was based on several assumptions that were not confirmed by the phase 3		

Drug	Indication	Nature of EMA concerns about study methods and findings as reported in EPAR	Information from sections 'What benefits of drug X have been shown in studies?' and "Why is drug X approved?' in Public Summaries	Information from section 'How does drug X work?' in PILs
		data…[T]here are substantial uncertainties regarding the efficacy of atezolizumab for the treatment of adult patients with locally advanced or metastatic urothelial carcinoma after prior chemotherapy or who are considered cisplatin ineligible, which are not outweighed by the likely favourable safety profile" (p202).		
Rucaparib (Rubraca)<br><br>Approved 23/05/2018<br><br>Conditional marketing authorisation	Treatment of platinum sensitive, relapsed or progressive, BRCA mutated, high-grade epithelial ovarian, fallopian tube, or primary peritoneal cancer, who have been treated with two or more prior lines of platinum based chemotherapy, and who are	Multiple concerns, including uncertainty over drug efficacy<br>Expert consultation:<br>"The [Scientific Advisory Group] disagreed on whether sufficient efficacy had been demonstrated… According to one (prevailing) view… there were no significant concerns about potential detrimental effects (albeit, this would have to be confirmed post-approval even if indirectly) and rucaparib represents an additional option for some patients… According to the opposing view, the methodological flaws (including selection bias, retrospective analysis,	"Two… studies looked at 106 patients with ovarian cancer and BRCA mutation whose cancer had come back following at least 2 previous cancer treatments, including in many cases treatment with platinum-based medicines. Of the 79 patients whose disease had responded in the past to platinum based medicines, 65% (51 patients) had a response to treatment with Rubraca and the response lasted on average 294 days (around 10 months)."	*'Rubraca blocks an enzyme that repairs damaged DNA in the cancer cells, resulting in their death."* (Emphasis added)

Drug	Indication	Nature of EMA concerns about study methods and findings as reported in EPAR	Information from sections 'What benefits of drug X have been shown in studies?' and "Why is drug X approved?' in Public Summaries	Information from section 'How does drug X work?' in PILs
	unable to tolerate further platinum based chemotherapy.	lack of convincing comparative data, lack of generalizability) preclude any conclusion about the efficacy of rucaparib compared to trabectedin+PLD. According this view, there are significant concerns about potential loss of efficacy in the absence of more definitive and direct comparative data. Furthermore, in many instances these patients would likely have been treated with PARP inhibitors in the maintenance setting making the target population somewhat theoretical. There were also concerns that patients could be misguided by apparent advantages in toxicity without due consideration to uncertainty about efficacy" (p.112).<br><br>Divergent opinion with EMA's Scientific Committee:<br>"Based on the currently available data we consider the benefit-risk balance for rucaparib in the proposed restricted indication to remain undetermined. Major uncertainties exist regarding the quality of the data,		

Drug	Indication	Nature of EMA concerns about study methods and findings as reported in EPAR	Information from sections 'What benefits of drug X have been shown in studies?' and "Why is drug X approved?' in Public Summaries	Information from section 'How does drug X work?' in PILs
		as the current pivotal results for the rucaparib application are derived from a post-hoc analysis from pooled data from two single arm trials involving only 106 patients. Also replication of the results was not provided. In the absence of a head-to-head comparison to standard of care, there are large uncertainties on the robustness and validity of the results. As a consequence, the current results cannot be contextualised and any reliable estimation of effect size is prevented. Although the activity of the drug in terms of response rate is noted, the assumed better toxicity profile as compared to standard of care does not outweigh the major uncertainties regarding efficacy. In conclusion, we cannot conclude on a positive relative [benefit/risk] and until more is known, the application is considered not approvable." (p167).		
**Neratinib** (Nerlynx)	Extended adjuvant	Concern about missing outcome data		"Nerlynx works by blocking the HER2

Drug	Indication	Nature of EMA concerns about study methods and findings as reported in EPAR	Information from sections 'What benefits of drug X have been shown in studies?' and "Why is drug X approved?' in Public Summaries	Information from section 'How does drug X work?' in PILs
Approved 31/08/2018<br><br>Regular marketing authorisation	treatment of patients with early-stage hormone receptor positive HER2-overexpressed/amplified breast cancer and who are less than one year from the completion of prior adjuvant trastuzumab based therapy.	"The study was originally planned with 5-year follow up for [invasive disease-free survival]... However, for commercial reasons, follow-up was truncated at two years of follow-up. Subsequently patients were reconsented for further follow-up to year five post baseline, where outcomes were captured retrospectively. Approximately 75% of patients were reconsented; furthermore, there was differential reconsent in the respective arms with more patients in the Nerlynx arm refusing reconsent" (p161).<br><br>"Sensitivity analyses provide some reassurance that the missing data is unlikely to affect the study conclusions. However, bias cannot be completely excluded" (p.144).<br><br><u>Uncertainty regarding magnitude of benefit</u><br>"The evidence of efficacy, from a single pivotal trial, is not compelling. The hazard ratio point estimate is 0.66	"Nerlynx has been shown to be more effective than placebo (a dummy treatment) at preventing the cancer from coming back in one main study involving 2,840 women with HER2-positive early breast cancer who had already received trastuzumab."<br><br>"Around 94% of the women given a year's treatment with Nerlynx lived for 1 further year after stopping Nerlynx without their cancer coming back versus 92% of those given placebo. When only women with hormone-receptor positive cancer were considered, about 95% of those given Nerlynx lived another year without the cancer coming back versus 91% of those given placebo."<br><br>"The European Medicines Agency considered that Nerlynx had been shown to be of benefit in women with HER2-positive early breast cancer, and that this benefit seemed to be mainly in women with hormone-receptor positive disease."	receptors on the cancer cells. *This helps to stop the cells from dividing and growing*."<br>(Emphasis added)

Drug	Indication	Nature of EMA concerns about study methods and findings as reported in EPAR	Information from sections 'What benefits of drug X have been shown in studies?' and "Why is drug X approved?' in Public Summaries	Information from section 'How does drug X work?' in PILs
		for 2-year [invasive disease-free survival]. This translates to an absolute 2-year treatment difference of 2.3%, which is modest, although of clinical relevance. The 95% confidence interval upper bound for the hazard ratio is 0.90, and the 2-sided p value is 0.008. Therefore, there remains uncertainty regarding the magnitude of the [invasive disease-free survival] benefit" (p118-9).<br><br><u>Uncertainty regarding clinical relevance of findings</u><br>"For the clinically relevant endpoint of [distant disease-free survival], statistical significance was not achieved… The effect of neratinib on [overall survival] is unknown. There is currently no evidence that the difference in [invasive disease-free survival] will translated to a survival benefit or that a detriment in terms of OS can be excluded" (p.144).<br><br>"There was a lack of strong support from clinically relevant secondary		

Drug	Indication	Nature of EMA concerns about study methods and findings as reported in EPAR	Information from sections 'What benefits of drug X have been shown in studies?' and "Why is drug X approved?' in Public Summaries	Information from section 'How does drug X work?' in PILs
		endpoints including distant disease-free survival" (p147).<br><br>Internal inconsistency in outcomes<br>"There is internal inconsistency in the outcomes, as the isolation of the measured effect to hormone receptor positive patients lacks a clear rationale, contributing to uncertainty" (p.147).<br><br>Expert consultation:<br>"Inconsistency between subgroups based on [hormone receptor] status in exploratory subgroup analyses lack convincing biological rationale and would need to be confirmed after long follow-up, 10 years or more for the Er+ subgroup" (p118).<br><br>Uncertain therapeutic value<br>Expert consultation:<br>"The views of the Scientific Advisory Group diverged". One view considered that "the small difference observed and high number-needed-to-treat is not considered a clear benefit; the intermediate endpoint of [invasive		

Drug	Indication	Nature of EMA concerns about study methods and findings as reported in EPAR	Information from sections 'What benefits of drug X have been shown in studies?' and "Why is drug X approved?' in Public Summaries	Information from section 'How does drug X work?' in PILs
		disease-free survival] includes local recurrences that are operable and do not represent a clearly worse prognosis; no data on [overall survival] have been presented and are very unlikely to ever be observed in this trial; no statistically significant effect has been demonstrated in terms of distant metastases; there is substantial uncertainty about the magnitude of the effect in view of the poor conduct of the study and unpredictable effect of censoring, both in the [invasive disease-free survival] and [quality of life] results. Concerning [invasive disease-free survival], the shape of the survival curve for neratinib shows a drop around the 2-year timepoint that is difficult to explain from a clinical point of view and is likely due to a data collection issue, introducing possible bias and further uncertainty about the magnitude of the effect. In conclusion, due to the limitations of the study and many remaining uncertainties, the efficacy cannot be		

Drug	Indication	Nature of EMA concerns about study methods and findings as reported in EPAR	Information from sections 'What benefits of drug X have been shown in studies?' and "Why is drug X approved?' in Public Summaries	Information from section 'How does drug X work?' in PILs
		considered convincingly demonstrated" (p118).<br><br><u>Divergent opinion within EMA's Scientific Committee:</u><br>"Although the single pivotal trial submitted in support of the application achieves nominal statistical significance, it falls short of the expectations to be exceptionally compelling and with precise estimates of the treatment effect as described in the points to consider document on applications with one pivotal study ... In addition the company's claim that there is substantially greater efficacy in the [hormone receptor positive] patients in not accepted. The data on the ER/HER cross-talk presented during the re-examination to support a possible mechanistic explanation for efficacy selectively in [hormone receptor positive] patients appeared plausible but must be considered hypothesis generating.... Neratinib causes significant gastrointenstinal toxicity. Diarrhoea affects most		

Drug	Indication	Nature of EMA concerns about study methods and findings as reported in EPAR	Information from sections 'What benefits of drug X have been shown in studies?' and "Why is drug X approved?' in Public Summaries	Information from section 'How does drug X work?' in PILs
		patients, is severe in a high proportion, and can be expected to affect quality of life… in the context of deficiencies in the efficacy demonstration, the adverse effect profile of neratinib is a matter of important concern" (p169).		
**Lorlatinib** (Lorviqua)<br><br>Approved 06/05/2019<br><br>Conditional marketing authorisation	Treatment of adult patients with ALK-positive advanced non-small cell lung cancer whose disease has progressed after: alectinib or ceitinib as the first ALK tyrosine kinase inhibitor ~~(TKI)~~ therapy; or crizotinib and at least one other ALK tyrosine kinase inhibitor	Uncertain therapeutic value<br>"The [overall response rate] by [Independent Review Committee] in the Phase 2 part was 42.9% (95%CI: 24.5-62.8) in EXP-3B and 39.6% (95%CI: 30.5-49.4) in EXP-4:EXP-5. In addition, approximately a third of the patients in both cohorts had stable disease. These results are not outstanding but within an expectable range, considering that the [overall response rate] for other ALK inhibitors are around 50% in the second-line setting and that the [overall response rate] generally drops down through the lines of therapy. The magnitude of this effect is difficult to interpret in the absence of	"Lorviqua was effective at treating ALK-positive NSCLC in one main study which included 139 patients… [a]round 43% of patients who had been previously treated with alectinib or ceritinib were considered by their doctors to have had a complete or partial response to the medicine. Out of patients who had been previously treated with crizotinib and another ALK tyrosine kinase inhibitor, around 40% had a complete or partial response to the medicine."<br><br>"Lorviqua was also effective when the cancer had spread to the brain. Depending on which previous treatment the patients had received,	"Lorviqua inhibits a type of enzyme called tyrosine kinase and *triggers the death of cancer cells* in patients with alterations in genes for ALK. Lorviqua is only given to patients whose disease is due to an alteration in the gene for ALK tyrosine kinase."<br>(Emphasis added)

Drug	Indication	Nature of EMA concerns about study methods and findings as reported in EPAR	Information from sections 'What benefits of drug X have been shown in studies?' and "Why is drug X approved?' in Public Summaries	Information from section 'How does drug X work?' in PILs
		a comparator; however, the ability to induce complete remission, although in a very small fraction of subjects, is noted" (p105).<br><br><u>Need for additional data in second line use</u><br>"Only 28 patients were included in the cohort EXP-3B including patients who have progressed after a second generation ALK inhibitors used as a first line treatment. As a consequence further data are needed to confirm the efficacy of lorlatinib in that setting and the applicant agreed to conduct a prospective observational single arm study to confirm the observed results from EXP-3B" (p107).<br><br><u>Divergent opinion within EMA's Scientific Committee</u><br>"Major therapeutic advantage has not been shown for the entire claimed indication, as efficacy in the second-line setting (i.e. after a previous treatment with a second-generation ALK tyrosine kinase inhibitor) is	around 67% and 52% of patients treated with Lorviqua had no signs of cancer in the brain or the signs of cancer had reduced."<br><br>"Lorviqua has been given 'conditional authorisation'. There is more evidence to come about the medicine, which the company is required to provide."	

Drug	Indication	Nature of EMA concerns about study methods and findings as reported in EPAR	Information from sections 'What benefits of drug X have been shown in studies?' and "Why is drug X approved?' in Public Summaries	Information from section 'How does drug X work?' in PILs
		currently not sufficiently established. The number of patients in the second line (n=28) does not allow for a proper evaluation of the efficacy data.<br>- The major therapeutic advantage shown in terms of efficacy in third or further lines cannot be extrapolated to the second-line setting, as the Applicant has not provided (non)-clinical data in support for such extrapolation approach." (p.148)		
**Talazoparib** (Talzenna)<br><br>Approved 20/06/2019<br><br>Regular marketing authorisation	Treatment of adult patients with germline BRCA1/2-mutations, who have HER2 negative locally advanced or metastatic breast cancer.	Concern over inappropriate comparator arm<br>The company had been advised by regulators "to include platinum cytotoxic treatment as one of the control arms (physician choice [of] treatment)… [This advice] was not followed." (p.99)<br><br>Expert consultation:<br>"The Scientific Advisory Group further noted that the control group of the pivotal clinical study excluded the use of a platinum-containing	"Talzenna was shown to be effective at increasing the time patients live without their disease getting worse in one main study involving 431 patients with HER2-negative breast cancer with BRCA mutations whose cancer had spread. Patients treated with Talzenna lived on average for 8.6 months without their disease getting worse compared with 5.6 months for patients treated with the doctor's choice of another cancer medicine."	"Patients with changes (mutations) in genes called BRCA are at risk of developing some forms of cancer. Talzenna works by blocking PARP, which is an enzyme that repairs damaged DNA in certain cancer cells. *As a result, the cancer cells can no longer repair themselves and they die.*"

Drug	Indication	Nature of EMA concerns about study methods and findings as reported in EPAR	Information from sections 'What benefits of drug X have been shown in studies?' and "Why is drug X approved?' in Public Summaries	Information from section 'How does drug X work?' in PILs
		regimen, which is considered more efficacious than the physician's choice monotherapies used in the pivotal trial. Thus, a smaller effect of PARP-inhibition would be expected compared to current standard treatments (although the toxicity profile is likely improved compared to platinum-containing regimen). Furthermore, the compliance in the physician's choice arm indicated problems. Whether a PARP-inhibitor is more efficacious than platinum-containing regimens in the population of gBRCA-associated metastatic breast cancer has not been established" (p.104).<br><br><u>Uncertainty over magnitude of treatment benefit due to missing outcome data</u><br>"The prolongation of [progression-free survival] compared with chemotherapy is modest, but the positive study results established talazoparib as an effective treatment		(Emphasis added)

Drug	Indication	Nature of EMA concerns about study methods and findings as reported in EPAR	Information from sections 'What benefits of drug X have been shown in studies?' and "Why is drug X approved?' in Public Summaries	Information from section 'How does drug X work?' in PILs
		within the scope of the indication" (p.138).<br><br>"Imbalances in withdrawal rates at baseline and prior to endpoints constitute the main uncertainties, both with respect to [progression-free survival] and [overall survival]. During the review, the applicant provided a number of sensitivity analyses indicating that the metrics are reasonably robust to assumptions of informative censoring. As the extent of bias that might be introduced cannot be precisely defined there is a residual uncertainty in the effect estimates due to the amount of missing data; however, the uncertainty and extent of potential bias, given the sensitivity analyses provided, is not large enough to question the beneficial effect of talazoparib" (p.135).		
**Larotrectinib** (Vitrakvi)	Treatment of adult and	<u>Uncertainties around clinical benefit across different tumour types</u>	"Three ongoing studies in 102 patients with solid tumours with NTRK gene	"In patients whose cancer is due to an

Drug	Indication	Nature of EMA concerns about study methods and findings as reported in EPAR	Information from sections 'What benefits of drug X have been shown in studies?' and "Why is drug X approved?' in Public Summaries	Information from section 'How does drug X work?' in PILs
Appoved 19/09/2019<br><br>Conditional marketing authorisation	paediatric patients with solid tumours that display a Neurotrophic Tyrosine Receptor Kinase (NTRK) gene fusion, who have a disease that is locally advanced, metastatic or where surgical resection is likely to result in severe morbidity; and who have no satisfactory treatment options	"The objective response rate was highly variable across the studied tumour types, from 0% [overall response rate] in single patients with breast cancer, cholangiocarcinoma and pancreatic cancer to 100% in the 4 patients with GIST. Tumour types where NTRK gene fusions are characteristic (or even considered pathognomonic) of the disease, such as Infantile fibrosarcoma (IFS, n=13), Salivary gland/MASC (n=10), and congenital mesoblastic nephroma (n=1), tended to have higher [overall response rate] (92%, 80%, and 100%, respectively). However, these estimates are not robust due to the small sample sizes of individual subgroups." (p146)<br><br>"As the NTRK fusion is rare in more common tumour types, there is still limited information on the level of efficacy in some otherwise common tumour types, such as non-secretory breast cancer, melanoma and colorectal cancer. A lower [overall	fusion showed that Vitrakvi is effective at reducing the size of patients' tumours. In these studies, 67% of patients who took Vitrakvi had a reduction in the size of their tumours, and the tumours on average shrank to less than half their original size. In addition, the tumours shrank quickly (within 2 months)."<br><br>"Vitrakvi differs from many other cancer medicines by targeting certain tumours with a specific gene arrangement wherever they occur in the body. Although studies are still underway, the results released so far show that it is effective at reducing the size of patients' tumours. In addition, the short time taken to shrink the tumours is important in relieving patients' symptoms."<br><br>"Vitrakvi has been given 'conditional authorisation'. This means that there is more evidence to come about the medicine, which the company is required to provide."	altered NTRK gene, the change in the gene causes the body to make an abnormal protein called TRK fusion protein, which can lead to uncontrolled cell growth and cancer. VITRAKVI blocks the action of TRK fusion proteins and so *may slow or stop the growth of the cancer. It may also help to shrink the cancer.*" (Emphasis added)

Drug	Indication	Nature of EMA concerns about study methods and findings as reported in EPAR	Information from sections 'What benefits of drug X have been shown in studies?' and "Why is drug X approved?' in Public Summaries	Information from section 'How does drug X work?' in PILs
		response rate] was observed in these histologies as well as a shorter duration of response compared to other responding tumour types but the numbers are too small to draw any conclusions." (p147)<br><br><u>Expert consultation:</u><br>"The Scientific Advisory Group agreed by consensus that available data do not support the hypothesis that NTRK gene fusions are universally oncogenic "drivers", independently of tumour type/histology and other disease characteristics; that the relevance of the mechanism of action may differ according to these and other characteristics; and that the non-clinical and clinical data are insufficient to establish activity regardless of tumour type and other characteristics… there are only few tumour types (listed above) for which NTRK fusions have been established as oncogenic "drivers" regardless of other characteristics… For other		

Drug	Indication	Nature of EMA concerns about study methods and findings as reported in EPAR	Information from sections 'What benefits of drug X have been shown in studies?' and "Why is drug X approved?' in Public Summaries	Information from section 'How does drug X work?' in PILs
		conditions, the role of NTRK fusions as oncogenic "drivers" is not properly studied and well-established. There are insufficient data to establish the activity of larotrectinib due to lack of comprehensive sequencing of tumour tissue prior to treatment, the small sample size in different tumour types, the significant heterogeneity observed in terms of [overall response rate] coupled with the notably very low [overall response rate] observed in different tumour types ([overall response rate]=0%-33%), especially in those common tumour types where occurrence of NTRK gene fusion is rare (lung, colon, breast)." (p.149)<br><br>"The proposed indication encompassing all solid tumours independently of tumour type does not reflect the conditions where efficacy has been established based on available data or reasonable extrapolations. In addition, however, one can note that larotrectinib has demonstrated an acceptable safety		

Drug	Indication	Nature of EMA concerns about study methods and findings as reported in EPAR	Information from sections 'What benefits of drug X have been shown in studies?' and "Why is drug X approved?' in Public Summaries	Information from section 'How does drug X work?' in PILs
		profile, albeit the number of exposed patients is low. In conclusion, evidence-based clinical decisions to use larotrectinib are only justified for the rare conditions listed above and in situations where established alternatives are lacking or, as in the case of major surgical procedures, where available alternatives are associated with high morbidity and mortality. From a clinical decision perspective, use of larotrectinib may be an attractive therapeutic option when established effective treatments are lacking and when based on relevant tumour tissue sequencing one could confirm the presence of NTRK-fusions and exclude other known oncogenic "drivers". However, such decisions cannot be considered evidence-based due to the lack of clinical evidence and the lack of predictive ability of clinical decision algorithms purely based on sequencing data. Nevertheless, such approaches warrant further investigation" (p.150).		

Drug	Indication	Nature of EMA concerns about study methods and findings as reported in EPAR	Information from sections 'What benefits of drug X have been shown in studies?' and "Why is drug X approved?' in Public Summaries	Information from section 'How does drug X work?' in PILs
		Uncertainty around estimates of benefit due to methodological deficiencies<br>"The application is considered lacking in prospectively studied cohorts that could provide an unbiased estimate of [overall response rate]. The uncertainty about the magnitude of the effect estimate due to these circumstances is of importance with respect to SmPC claims, and further strengthens the case for the non-comprehensiveness of available data. A prospective cohort is required to produce an unbiased estimate of efficacy" (p188).<br><br>"The non-randomised design further hampers the assessment of particularly the time-dependent outcomes. The small efficacy data base raises issues with regard to the representativeness in relation to the indication sought, encompassing any solid tumour type. This aspect will be to some extent addressed during the post-marketing study and external controls. This		

Drug	Indication	Nature of EMA concerns about study methods and findings as reported in EPAR	Information from sections 'What benefits of drug X have been shown in studies?' and "Why is drug X approved?' in Public Summaries	Information from section 'How does drug X work?' in PILs
		uncertainty is stated in the SmPC" (p188).<br><br>"Due to the small sample size, the confidence intervals are generally wide, making efficacy estimates generally imprecise and hampering the possibility to draw conclusions regarding efficacy in subgroups, e.g. with regard to age groups and gene fusion type. This aspect will be addressed by the post-marketing study)" (p188).<br><br>"Overall, notwithstanding the considerable methodological caveats outlined above, the efficacy estimates available today may be considered outstanding in this generally late stage disease setting. The main issue efficacy-wise is the robustness and generalisability of these estimates. While it is likely that the estimates may change, possibly in a negative direction, the present outstanding estimates provide some reassurance as to the presence of a large treatment		

Drug	Indication	Nature of EMA concerns about study methods and findings as reported in EPAR	Information from sections 'What benefits of drug X have been shown in studies?' and "Why is drug X approved?' in Public Summaries	Information from section 'How does drug X work?' in PILs
		benefit. Important quantitative interactions between treatment and tumour type will be further explored." (p152)<br><br>Re: pooled data "Issues of multiplicity and bias due to the adaptive design cause the efficacy outcomes likely to be inflated."(p190)<br><br>Overall, "Although there is uncertainty about the precise magnitude of effect, both due to the study conduct, and since the understanding of the extent that tissue of origin is an effect modifier is incomplete, the observed [overall response rate] of 72% is considered outstanding. Also when the [overall response rate] results divided into adult (68%) and paediatric (82%) patients, results in both subgroups are considered outstanding" (p191).<br><br>"Notwithstanding this, the explorative and adaptive nature of the study program, the immaturity of the		

Drug	Indication	Nature of EMA concerns about study methods and findings as reported in EPAR	Information from sections 'What benefits of drug X have been shown in studies?' and "Why is drug X approved?' in Public Summaries	Information from section 'How does drug X work?' in PILs
		[duration of response] data, the single arm nature of the studies, as well as the limitations of the data with respect to understanding the extent that tissue origin might act as an effect modifier, available data are considered non-comprehensive" (p192).<br><br><u>Uncertainty associated with the clinical relevance of study findings for patients</u><br>"[Progression-free survival] and [overall survival] are important for contextualisation of the [overall response rate] and [duration of response] results and in relation to approved anticancer products normally approved based on [progression-free survival] and/or [overall survival]. However, due to the pooling of many different types of primary malignancies with inherently different prognosis, the data should be interpreted with caution" (p.146).<br><br>"The interpretation of [patient-reported outcomes] from single-arm		

Drug	Indication	Nature of EMA concerns about study methods and findings as reported in EPAR	Information from sections 'What benefits of drug X have been shown in studies?' and "Why is drug X approved?' in Public Summaries	Information from section 'How does drug X work?' in PILs
		open-label studies is generally difficult, due to the non-blinded study design's effect on the patients' experience and the lack of comparator. In the present case, also lack of formal hypothesis testing and the missing data preclude the acceptance of any [health-related quality-of-life] claims in the SmPC" (p126).<br><br>Need for additional data<br>"- The benefit in subgroups of patients based on histology. This may be studied in terms of [overall response rate] and [duration of response] in prospective single-arm studies encompassing a broad variety of tumour types.<br>- The requirement for unbiased estimate of [overall response rate] and [duration of response]. This may be studied in a prospective cohort study" (p151).		
**Examples where concerns related to at least one source of uncertainty are accurately reported in the Public Summary, but not the PIL** *(Relevant sentences* ***in bold****)*				

**Drug**	**Indication**	**Nature of EMA concerns about study methods and findings as reported in EPAR**	**Information from sections 'What benefits of drug X have been shown in studies?' and "Why is drug X approved?' in Public Summaries**	**Information from section 'How does drug X work?' in PILs**
**Avelumab** (Bavencio)<br><br>Approved 18/09/2017<br><br>Conditional marketing authorisation	Treatment of adult patients with metastatic Merkel cell carcinoma ~~(MCC)~~	Lack of comparator arm<br>"As the 003 study was designed as a single arm trial, there is no comparator arm to determine the true effect size observed for avelumab in terms of [overall response rate], [duration of response] and [progression-free survival] for both chemotherapy-treated and naive patients" (p120).<br><br>Small treatment effects in second line<br>"The reported [overall response rate] (33%) for avelumab in the next-line treatment of [metastatic Merkel cell carcinoma] is not considered outstanding, however, durability of responses is convincing" (p124).<br><br>Need for additional data in first line<br>"…although the data in 1st line was promising, the data is limited as few patients have reached the 6 month milestone and further confirmatory data from additional treated patients would be needed to confirm the effect size of the benefit" (p89, also see p90).	"In a main study involving 88 patients with metastatic MCC who had received previous treatment with chemotherapy (cancer medicines), around 33% of patients (29 out of 88) were considered to have had a reduction in tumour size or no sign of it after treatment with the medicine; in most of these patients, the response lasted for at least 6 months."<br><br>"Early results from an ongoing study looking at the effects of Bavencio in patients with metastatic MCC who had not received previous chemotherapy showed that the overall response rate at the time of analysis was 62% (18 out of 29 patients)."<br><br>"Patients with MCC that has spread and come back after initial treatment with chemotherapy have very limited treatment options. **Although the response rates to Bavencio are not outstanding,** the duration of the response (at least 6 months) is	"PD-L1 is found on the surface of MCC cells, and helps protect tumour cells from the immune system (the body's natural defences). Bavencio binds to PD-L1, and blocks this protective effect, *allowing the immune system to attack the tumour cells*." (Emphasis added)

Drug	Indication	Nature of EMA concerns about study methods and findings as reported in EPAR	Information from sections 'What benefits of drug X have been shown in studies?' and "Why is drug X approved?' in Public Summaries	Information from section 'How does drug X work?' in PILs
		Divergent opinion within EMA's Scientific Committee<br>"The evidence available to date regarding efficacy and safety of Bavencio (avelumab) in the chemotherapy-naïve [metastatic Merkel cell carcinoma] population is considered insufficient to support a positive benefit/risk. Current data on the treatment-naïve [metastatic Merkel cell carcinoma] patients from the pivotal study EMR100070-003 (Cohort B) indicate an [overall response rate]of 71% in 14 patients with at least 6 months follow up and an overall response rate] of 65% in the 29 pts with at least 13 weeks follow-up. From these data no superiority in overall response rate] can be concluded when compared to chemotherapy (overall response rate] up to 70%). The median [duration of response] of chemotherapy is 6 months, however, the [duration of response] in Bavencio-treated patients is immature and early evidence of	important for these patients, since the responses seen with chemotherapy medicines are of shorter duration."<br><br>"Since Bavencio has been granted a conditional approval for MCC, the company that markets the medicine will provide further data from the ongoing study of patients who did not receive chemotherapy before starting treatment with Bavencio."	

Drug	Indication	Nature of EMA concerns about study methods and findings as reported in EPAR	Information from sections 'What benefits of drug X have been shown in studies?' and "Why is drug X approved?' in Public Summaries	Information from section 'How does drug X work?' in PILs
		durability is observed in only 6 subjects reported to have a [duration of response] of at least 6 months. Further concerns are raised over the very small number of patients enrolled in Part-B of Study 003 (n=29/112) and the limited follow-up duration taking into consideration that alternative treatment options (i.e., chemotherapy) are available with relatively high rates of response" (p.130).		
**Padeliporfin** (Tookad)<br><br>Approved 10/11/2017<br><br>Regulator marketing authorisation	Previously untreated, unilateral, low-risk, adenocarcinoma of the prostate with life expectancy ≥ 10 years	<u>Uncertainty associated with assessment of trial endpoint</u><br>"The duration of biopsy follow-up in the trial was insufficient to accurately determine time-to-progression. It is notes that this is histological rather than biochemical in progression as reported for other treatment modalities and the correlation between these two measures is unclear" (p.91).	"A study of 413 men with low-risk prostate cancer found that Tookad with VTP therapy was effective at clearing signs of prostate cancer in many patients. After 24 months, 49% of patients treated with Tookad had no definitive signs of cancer in their tissues compared with 14% of patients who received no treatment."<br><br>"In addition, Tookad helped delay the progression from low-risk prostate cancer to a higher risk cancer. On	Once it has been given, TOOKAD has to be activated by laser light shone along a fibre that targets the light onto the cancer. *The activated medicine then causes the death of the cancer cells*." (Emphasis added)<br><br>"To date information beyond two years after VTP procedure is

Drug	Indication	Nature of EMA concerns about study methods and findings as reported in EPAR	Information from sections 'What benefits of drug X have been shown in studies?' and "Why is drug X approved?' in Public Summaries	Information from section 'How does drug X work?' in PILs
		Need for longer-term data and uncertainty around therapeutic value of drug<br>"It is… uncertain whether Tookad [vascular targeted photdynamic therapy] (VTP) adversely influences the ability to later undertake radical therapy, either 'missed opportunity' through disease progression or due to the PD effect of vascular occlusion. There is concern that associated fibrosis may make surgery more difficult and impact post-operative wound healing although the limited available data does not indicate greater difficulty to perform radical prostatectomy" (p139).<br><br>Expert consultation:<br>"Tookad VTP is claimed to primarily defer rather than avoid the need for radical therapy... and in the short term there seemed to be a positive benefit-risk balance. However, in the context of low-risk prostate cancer, with active surveillance being a valid management option and the existence of effective	average progression occurred after 28 months in patients treated with Tookad compared with 14 months in those who did not receive treatment."<br><br>"**It is unclear… what effect Tookad treatment will have on future treatments for prostate cancer and more study is needed to find out if such treatments are compromised**."	limited and so, at this time, **data are currently not available to know whether the benefit of TOOKAD-VTP is long-lasting**. If you do require further treatment, **at the moment, there is limited information on whether TOOKAD-VTP affects the efficacy and safety results of other treatments (such as surgery to remove the prostate or radiotherapy)."**

Drug	Indication	Nature of EMA concerns about study methods and findings as reported in EPAR	Information from sections 'What benefits of drug X have been shown in studies?' and "Why is drug X approved?' in Public Summaries	Information from section 'How does drug X work?' in PILs
		treatments in case of progression, it is the long-term benefits (and risks), which bear more weight in the overall benefit-risk assessment… [T]he follow-up of available studies with Tookad VTP is of insufficient duration to understand the long-term effects given the complex and heterogeneous prostate cancer biology requiring 10 or more years of follow-up. Without this information, it is impossible to make informed treatment decisions… For a majority of Scientific Advisory Group members, this risk was considered unjustified, in the absence of efficacy data showing at least non-inferiority of the approach in terms of long-term outcome. Given the existence of highly effective alternative therapy (radical therapy) and the fact that the disease is not rare, a risk of compromising long-term outcome was not considered justified" (p97).<br><br><u>Divergent opinion within EMA"s Scientific Committee:</u>		

Drug	Indication	Nature of EMA concerns about study methods and findings as reported in EPAR	Information from sections 'What benefits of drug X have been shown in studies?' and "Why is drug X approved?' in Public Summaries	Information from section 'How does drug X work?' in PILs
		"Although a short term benefit in terms of progression of the disease has been shown in study PCM301, the clinical relevance remains to be established… [T]he efficacy data are not sufficient to support at least non-inferiority in terms of long-term outcomes… Importantly, there is virtually no data on the potential consequences of post-Tookad local scarring in case of disease progression. Thus the safety and efficacy of subsequent radical therapy is uncertain and it still needs to be demonstrated that treatment with Tookad does not compromise the eligibility for and the results of subsequent radical therapy" (p145).		
**Rucaparib** (Rubraca)<br><br>Approved 23/05/2018	Treatment of patients with platinum sensitive, relapsed or progressive, BRCA mutated, high grade epithelial ovarian,	<u>Multiple methodological deficiencies Expert consultation</u>:<br>"Due to the remaining uncertainty, the Scientific Advisory Group views diverged on whether efficacy had been sufficiently established… [According to one view] the methodological flaws (including selection bias, retrospective	"Two… studies looked at 106 patients with ovarian cancer and BRCA mutation whose cancer had come back following at least 2 previous cancer treatments, including in many cases treatment with platinum-based medicines. Of the 79 patients whose disease had responded in the past to	"Patients with changes (mutations) in genes called BRCA are at risk of developing a number of types of cancer. *Rubraca blocks an enzyme that repairs damaged*

Drug	Indication	Nature of EMA concerns about study methods and findings as reported in EPAR	Information from sections 'What benefits of drug X have been shown in studies?' and "Why is drug X approved?' in Public Summaries	Information from section 'How does drug X work?' in PILs
Conditional marketing authorisation	fallopian tube, or primary peritoneal cancer, who have been treated with two or more prior lines of platinum based therapy and who are unable to tolerate further platinum based therapy.	analysis, lack of convincing comparative data, lack of generalisability) preclude any conclusion about the efficacy of rucaparib compared to trabectedin+PLD… [T]here are significant concerns about potential loss of efficacy in the absence of more definitive and direct comparative data. Furthermore, in many instances these patients would likely have been treated with PARP inhibitors in the maintenance setting making the target population somewhat theoretical" (p112).<br><br><u>Divergent opinion within EMA's scientific committee:</u><br>"Based on the currently available data we consider the benefit-risk balance for rucaparib in the proposed restricted indication to remain undetermined. Major uncertainties exist regarding the quality of the data, as the current pivotal results for the rucaparib application are derived from a post-hoc analysis from pooled data	platinum based medicines, 65% (51 patients) had a response to treatment with Rubraca and the response lasted on average 294 days (around 10 months)."<br><br>"The European Medicines Agency decided that although **further study was needed to better understand the size of the benefit,** Rubraca's benefits are greater than its risks and it can be authorised for use in the EU."<br><br>"Rubraca has been given 'conditional authorisation'. This means that there is more evidence to come about the medicine, which the company is required to provide."	*DNA in the cancer cells, resulting in their death*." (Emphasis added)

Drug	Indication	Nature of EMA concerns about study methods and findings as reported in EPAR	Information from sections 'What benefits of drug X have been shown in studies?' and "Why is drug X approved?' in Public Summaries	Information from section 'How does drug X work?' in PILs
		from two single arm trials involving only 106 patients. Also replication of the results was not provided. In the absence of a head-to-head comparison to standard of care, there are large uncertainties on the robustness and validity of the results. As a consequence, the current results cannot be contextualised and any reliable estimation of effect size is prevented." (p167)		
**Ropeginterferon alfa-2b** (Besremi**)**<br><br>Approved 15/02/2019<br><br>Regular marketing authorisation	Treatment of polycythaemia vera without symptomatic splenomegaly	<u>Failure to demonstrate efficacy</u><br>"The pivotal PROUD-PV Study failed to show superiority as well as non-inferiority with respect to the pre-specified primary efficacy endpoint outcome "disease response rate at end of study (12 month)" (i.e. complete haematological response and spleen size normality). Post-hoc the applicant has proposed a redefined primary endpoint "complete haematological response (without spleen normalisation)" in order to demonstrate efficacy. ... Only for the new post-hoc primary endpoint	Besremi is effective at reducing levels of red blood cells in patients with polycythaemia vera. In this study, 43% of patients receiving Besremi had normal red blood cell counts after one year of treatment; 46% of patients receiving another medicine, hydroxycarbamide, had similar improvements.<br><br>Besremi is effective at reducing the excessive number of blood cells in patients with polycythaemia vera, and the proportion of patients improving increased with longer treatment.	"Interferons are produced by your immune system *to block the growth of cancer cells*." (Emphasis added)

Drug	Indication	Nature of EMA concerns about study methods and findings as reported in EPAR	Information from sections 'What benefits of drug X have been shown in studies?' and "Why is drug X approved?' in Public Summaries	Information from section 'How does drug X work?' in PILs
		analysis and post-hoc widened [non-inferiority] margin of – 20% non-inferiority could efficacy be demonstrated for ropeginterferon alfa-2b as indicated by a complete haematological response of 43.1% (43/122) in the ropeginterferon alfa-2b arm versus 45.6% (57/123) in the [hydroxycarbamide] arm after 12 months of treatment. The p-value is reported with 0.0028. However, this non-inferiority analysis can hardly be considered confirmatory at least from a methodological point of view" (p.83).<br><br>"[N]either a statistical nor a clinical justification of the -10.5% [non-inferiority]or -20% [non-inferiority]margin was provided. Therefore, from a biostatistical point of view, the actual differences between treatments as well as the results for the additional endpoints need to be interpreted in an exploratory sense only" (p82).	**Although Besremi may be less effective than hydroxycarbamide in the first months of treatment**, phlebotomy (a procedure to remove excess blood from the body) can help to control the condition in the short term.	

Drug	Indication	Nature of EMA concerns about study methods and findings as reported in EPAR	Information from sections 'What benefits of drug X have been shown in studies?' and "Why is drug X approved?' in Public Summaries	Information from section 'How does drug X work?' in PILs
		Expert consultation:<br>"[T]he Scientific Advisory Group considered that the pivotal study (PROUD-PV) suffered of critical deficiencies in the design and analysis, making it impossible to formally establish non-inferiority of Besremi compared to [hydroxycarbamide] at any time point. ... Similarly, any claims about the observed non-inferiority of Besremi at later time points remain to be established given the potential selection bias and lack of pre-specification and handling of multiplicity." (p84-5)<br><br>Uncertain therapeutic value<br>Expert consultation:<br>"A potential loss of 20% [because of the non-inferiority margin] or more in [complete hematologic response] is considered clinically significant, especially in the initial treatment where [hydroxycarbamide] is preferred to achieve a rapid response. However, given the availability of phlebotomy, this loss in the short term (<12		

Drug	Indication	Nature of EMA concerns about study methods and findings as reported in EPAR	Information from sections 'What benefits of drug X have been shown in studies?' and "Why is drug X approved?' in Public Summaries	Information from section 'How does drug X work?' in PILs
		months) is considered acceptable ... the benefits and risks associated with Besremi need to be clearly communicated to patients and physicians. Besremi should be indicated in patients for whom agents with higher activity are not preferred based on an informed clinical decision. It should also be made clear that inferior efficacy compared to [hydroxycarbamide] after 12 months has not been formally established and that patients are likely at higher risk of thrombotic events during the first 12 months (even if the trial did not show clear harms)" (p85).<br><br>"Scientific Advisory Group agreed that the indication should be restricted to patients with PV without splenomegaly for whom treatment with established active agents like [hydroxycarbamide] or ruxolitinib (where applicable) is not considered acceptable ..." (p85-6).		
